# Why do water quality monitoring programs succeed or fail? A qualitative comparative analysis of regulated testing systems in sub-Saharan Africa

**DOI:** 10.1016/j.ijheh.2018.05.010

**Published:** 2018-07

**Authors:** Rachel Peletz, Joyce Kisiangani, Mateyo Bonham, Patrick Ronoh, Caroline Delaire, Emily Kumpel, Sara Marks, Ranjiv Khush

**Affiliations:** a The Aquaya Institute, PO Box 21862−00505, Nairobi, Kenya; b Department of Sanitation, Water and Solid Waste for Development (Sandec), Eawag, Ueberlandstrasse 133, CH-8600, Duebendorf, Switzerland; c The Aquaya Institute, P.O. Box 5502, Santa Cruz, CA, 95063, United States

**Keywords:** Water quality, Water testing, Monitoring, Institutional capacity, Qualitative comparative analysis

## Abstract

**Background:**

Water quality testing is critical for guiding water safety management and ensuring public health. In many settings, however, water suppliers and surveillance agencies do not meet regulatory requirements for testing frequencies. This study examines the conditions that promote successful water quality monitoring in Africa, with the goal of providing evidence for strengthening regulated water quality testing programs.

**Methods and findings:**

We compared monitoring programs among 26 regulated water suppliers and surveillance agencies across six African countries. These institutions submitted monthly water quality testing results over 18 months. We also collected qualitative data on the conditions that influenced testing performance via approximately 821 h of semi-structured interviews and observations. Based on our qualitative data, we developed the Water Capacity Rating Diagnostic (WaterCaRD) to establish a scoring framework for evaluating the effects of the following conditions on testing performance: *accountability*, *staffing*, *program structure, finances*, and *equipment & services*. We summarized the qualitative data into case studies for each of the 26 institutions and then used the case studies to score the institutions against the conditions captured in WaterCaRD. Subsequently, we applied fuzzy-set Qualitative Comparative Analysis (fsQCA) to compare these scores against performance outcomes for water quality testing. We defined the performance outcomes as the proportion of testing Targets Achieved (outcome 1) and Testing Consistency (outcome 2) based on the monthly number of microbial water quality tests conducted by each institution. Our analysis identified *motivation & leadership*, *knowledge*, *staff retention*, and t*ransport* as institutional conditions that were necessary for achieving monitoring targets. In addition, *equipment, procurement, infrastructure,* and *enforcement* contributed to the pathways that resulted in strong monitoring performance.

**Conclusions:**

Our identification of institutional commitment, comprising *motivation & leadership*, *knowledge*, and *staff retention*, as a key driver of monitoring performance was not surprising: in weak regulatory environments, individuals and their motivations take-on greater importance in determining institutional and programmatic outcomes. Nevertheless, efforts to build data collection capacity in low-resource settings largely focus on supply-side interventions: the provision of infrastructure, equipment, and training sessions. Our results indicate that these interventions will continue to have limited long-term impacts and sustainability without complementary strategies for motivating or incentivizing water supply and surveillance agency managers to achieve testing goals. More broadly, our research demonstrates both an experimental approach for diagnosing the systems that underlie service provision and an analytical strategy for identifying appropriate interventions.

## 1 Introduction

Poor access to safe drinking water is a major cause of disease and death, particularly among young children in low-income countries. Limited supplies of drinking water and high levels of contamination are estimated to cause over 500,000 deaths per year from diarrheal disease alone; additional health concerns associated with unsafe drinking water include viral and parasitic infections, enteric dysfunction, growth faltering, and chemical toxicities (WHO, 2014; Prüss-Ustün et al., 2014; Liu et al., 2012).

Consequently, information about drinking water quality is essential for guiding efforts to reduce waterborne illnesses: accurate water quality data identifies high-risk water sources, determines effective water treatment methods, and contributes to the evaluation of water and sanitation improvement programs. This importance of water quality data is reflected in the framework that is proposed by the WHO/UNICEF Joint Monitoring Programme (JMP) to measure progress toward the United Nations’ post-2015 Sustainable Development Goals (SDGs) for drinking water, namely SDG target 6.1, which specifies universal and equitable access to safe and affordable drinking water for all by 2030 (WHO/UNICEF, 2017).

In most countries, regulations for managing drinking water safety also specify two complementary water quality testing activities: (1) operational (or process) monitoring by licensed water suppliers; and (2) surveillance (or compliance) monitoring by an independent agency, usually responsible for public health (Rahman et al., 2011; WHO, 2011). Operational monitoring verifies the effectiveness of treatment and distribution processes and guides corrective actions. Surveillance monitoring includes oversight of regulated water supplies and the assessment of informal and community managed water sources (WHO, 2011).

Despite these established responsibilities for monitoring drinking water quality, water suppliers and surveillance agencies often do not meet regulatory requirements for testing frequencies (the number of tests conducted within a defined time period), which we refer to as water quality monitoring performance. In a previous study of 72 regulated water suppliers and surveillance agencies across 10 sub-Saharan African countries, we found that 85% conducted some microbial water quality testing, yet, only 41% achieved the testing frequencies specified by national standards (Peletz et al., 2016). Water suppliers (all of which operated in urban settings) were more likely than surveillance agencies (which were primarily active in rural areas) to comply with testing requirements. Among both suppliers and surveillance agencies, larger operations (as determined by numbers of people served, annual water quality budget, and jurisdictions at national or regional levels) were positively associated with monitoring performance (Peletz et al., 2016). In contrast, the numbers of water quality staff per population served, years in operation, independent regulation, and documented national standards were not significantly associated with performance (Peletz et al., 2016).

Other studies have identified constraints to water quality testing in low-income settings, which include poor regulatory enforcement and insufficient resources for the personnel, equipment and logistical requirements of operating water quality testing programs (Lloyd and Helmer, 1991; Steynberg, 2002; Lloyd et al., 1987). These findings underlie recommendations for strengthening regulatory enforcement and ensuring financial resources (Rahman et al., 2011; Steynberg, 2002). Additional recommendations for strengthening water safety management include increased reliance on audit-based surveillance in urban areas serviced by regulated water suppliers, and the application of Water Safety Plans to mitigate water quality risks (Rahman et al., 2011; Lloyd and Bartram, 1991; WHO, 2011).

Currently, interventions for improving water quality monitoring performance among water suppliers and surveillance agencies tend to emphasize hardware and knowledge inputs, including upgrading laboratories, supplying equipment, introducing mobile phone applications for data management, and training personnel (US EPA, 2017; African Water Association, 2017; American Chemical Society, 2015; Mistry and Lawson, 2017). In addition, multiple efforts have focused on the development of appropriate testing methods for low-resource settings (Stauber et al., 2012; Bain et al., 2012; Rahman et al., 2010).

The effectiveness of these various recommendations and interventions, however, is rarely tested, and there is limited understanding of the institutional characteristics (termed ‘conditions’ throughout this paper) that influence water quality monitoring performance. We hypothesized that institutional monitoring performance depends on a range of conditions that extend beyond hardware, training, and financial resources. To test our hypothesis, we applied Qualitative Comparative Analysis (QCA) methods to evaluate the relationships between multiple institutional conditions and microbial water quality testing performance among 26 water suppliers and surveillance agencies across six African countries.

QCA compares cases (e.g., institutions), using both qualitative and quantitative methods, to determine which conditions or combinations of conditions explain variations in outcomes of interest (Jordan et al., 2011, 2016; Rihoux and Ragin, 2008; McAdam et al., 2010). QCA, which is appropriate for an intermediate sample size (5–50 cases), falls between in-depth detailed case studies and large quantitative studies designed to build multivariate statistical models of average effects (McAdam et al., 2010; Kunz et al., 2015; Rihoux and Ragin, 2008). QCA is increasingly used in the water and sanitation sector (Kaminsky and Jordan, 2017): for example, to examine the conditions that influence sanitation infrastructure sustainability (Kaminsky and Javernick-will, 2014), water utility recycling (Kunz et al., 2015), school sanitation management (Chatterley et al., 2014, 2013), rural water supply system sustainability (Marks et al., 2018), and water resources management (Huntjens et al., 2011; Srinivasan et al., 2012). To our knowledge, this is the first study to apply QCA to assess the conditions that influence the performance of water quality monitoring programs. Our research objective was to provide evidence that promotes effective strategies for strengthening regulated water quality testing programs.

## 2 Materials and methods

### 2.1 Study context: Monitoring for Safe Water (MfSW)

This study was conducted under The Aquaya Institute’s (Aquaya’s) Monitoring for Safe Water (MfSW) research program, which studies water safety monitoring and management by regulated agencies (Peletz et al., 2016, 2013). It is important to note that the research context of the MfSW program likely influenced some of the institutional conditions and associations being studied; these program effects are specified in the Discussion section. Water suppliers and surveillance agencies that collaborated with this MfSW study established their targets for microbial water quality testing frequencies according to both government standards and local management needs (e.g., Ugandan national standards specify a minimum of one sample per month for piped systems serving <2500 people). The MfSW program provided the collaborating institutions with the following financial inputs:

1) *Upfront capacity building grants* to fund additional testing equipment, trainings, and other scale-up costs, provided from May–November 2013.
2) *Per-test reward payments* for each microbial test conducted above baseline levels up to the institution’s monthly target. These payments ranged from 5 to 30 USD per test, depending on estimated testing costs, and were provided on a monthly basis from July 2013–December 2014 (the start dates for per-test reward payments varied by institution).
3) *Bonus Payments* if MfSW institutions met testing targets from July 2013–December 2014.

We structured these grants and incentives, to lower financial barriers to better monitoring performance, and, thereby, facilitate the identification of non-financial constraints. We also calibrated the financial packages according to each collaborating institution’s testing responsibilities: i.e., institutions required to test more water samples were eligible for larger grants and incentive payments. The amounts that institutions were eligible to receive ranged from USD 12,542 to 77,272.

We determined the amounts of the upfront grants based on both budget requests provided by the collaborating institutions and needs assessments that we conducted during on-site visits; we did not impose any requirements regarding the use of the per-test or bonus payments. The MfSW collaborating institutions were responsible for designing and managing their expanded microbial water quality testing programs, including: selection of testing methods; procurement of testing equipment; organization of training events; determination of sampling schedules and locations; coordination of sample collection and testing; management and analyses of data; and reporting to regulatory agencies and other stakeholders.

The collaborating institutions employed a variety of microbial detection methods, including: presence/absence tests (Hydrogen Sulfide (H_2_S) or *Escherichia. coli* (*E. coli*) assays in 100 mL or 10 mL sample volumes); membrane filtration-derived colony counts (*E. coli* and thermotolerant coliforms in 100 mL sample volumes); multiple test tube-derived most probable number estimates (thermotolerant and total coliforms in 100 mL sample volumes); and direct plate counts (*E. coli* in 1 mL sample volumes) (Peletz et al., 2016; Kumpel et al., 2016) (Table 1).

**Table 1:** Descriptions of MfSW collaborating institutions.

Case	Country	Supplier or surveillance agency	Description	Districts or networks covered by MfSW	Population in covered districts/ networks	Urban / Rural	Baseline tests (monthly)	Target tests (monthly)	Microbial testing method
E1	Ethiopia	SA	Regional surveillance agency	200 districts	20,532,125	R	8	185	MPN
E2	Ethiopia	SA	Regional surveillance agency	119 districts	7,330,967	R	1	71	MF
E3	Ethiopia	S	City supplier	1 network	410,000	U	1	51	MF
E4	Ethiopia	S	City supplier	1 network	13,900	U	0	15	MF
G1	Guinea	S	National supplier	5 networks	2,733,117	U	48	204	MF
K1	Kenya	S	City supplier	8 networks	137,060	U	0	25	PF + C
K2	Kenya	S	City supplier	1 network	400,000	U	41	82	MPN
K3	Kenya	S	City supplier	1 network	23,493	U	0	26	MF
K4	Kenya	S	City supplier	2 networks	90,000	U	9	28	MPN
K5	Kenya	SA	District health office	1 district	261,876	R	0	579	PA H2S
K6	Kenya	SA	District health office	1 district	177,572	U	29	332	MF
K7	Kenya	SA	District health office	1 district	608,649	R	0	87	PF + C
S1	Senegal	SA	National health ministry	13 districts	3,106,566	U	0	222	MF
U1	Uganda	S	National supplier	5 networks	291,769	U	79	200	MF
U2	Uganda	S	Private water operators association	15 networks	436,811	U	0	103	MF
U3	Uganda	SA	Regional surveillance agency	64 networks	3,133,638	R	100	392	MF
U4	Uganda	SA	District health office	1 district	774,800	R	1	84	MF
U5	Uganda	SA	District health office	1 district	239,878	R	5	146	MF
U6	Uganda	SA	District health office	1 district	478,192	R	0	78	MF
U7	Uganda	SA	District health office	1 district	358,239	R	0	190	MF
Z1	Zambia	S	Regional supplier	8 networks	87,717	U	85	204	MF
Z2	Zambia	S	Regional supplier	7 networks	37,716	R	18	53	MF
Z3	Zambia	SA	District health office	1 district	371,389	R	9	72	MF
Z4	Zambia	SA	District health office	1 district	469,666	R	35	88	PA H2S
Z5	Zambia	SA	District health office	1 district	2,011,957	U	41	125	MF
Z6	Zambia	SA	District health office	1 district	75,343	R	0	30	MF

U = urban; R = rural.

S = Supplier; SA = surveillance agency.

MF = membrane filtration; MPN = Most probable number; PF + C=Petrifilm and 10 mL Colilert presence-absence; PA H2S = presence absence hydrogen sulfide test.

All institutions using MF tested for thermotolerant coliforms; G1 also tested for *E.Coli*. All institutions using MPN tested for *E.coli*; E1 also tested for thermotolerant coliforms.

### 2.2 Study design

**Fig. 1:**
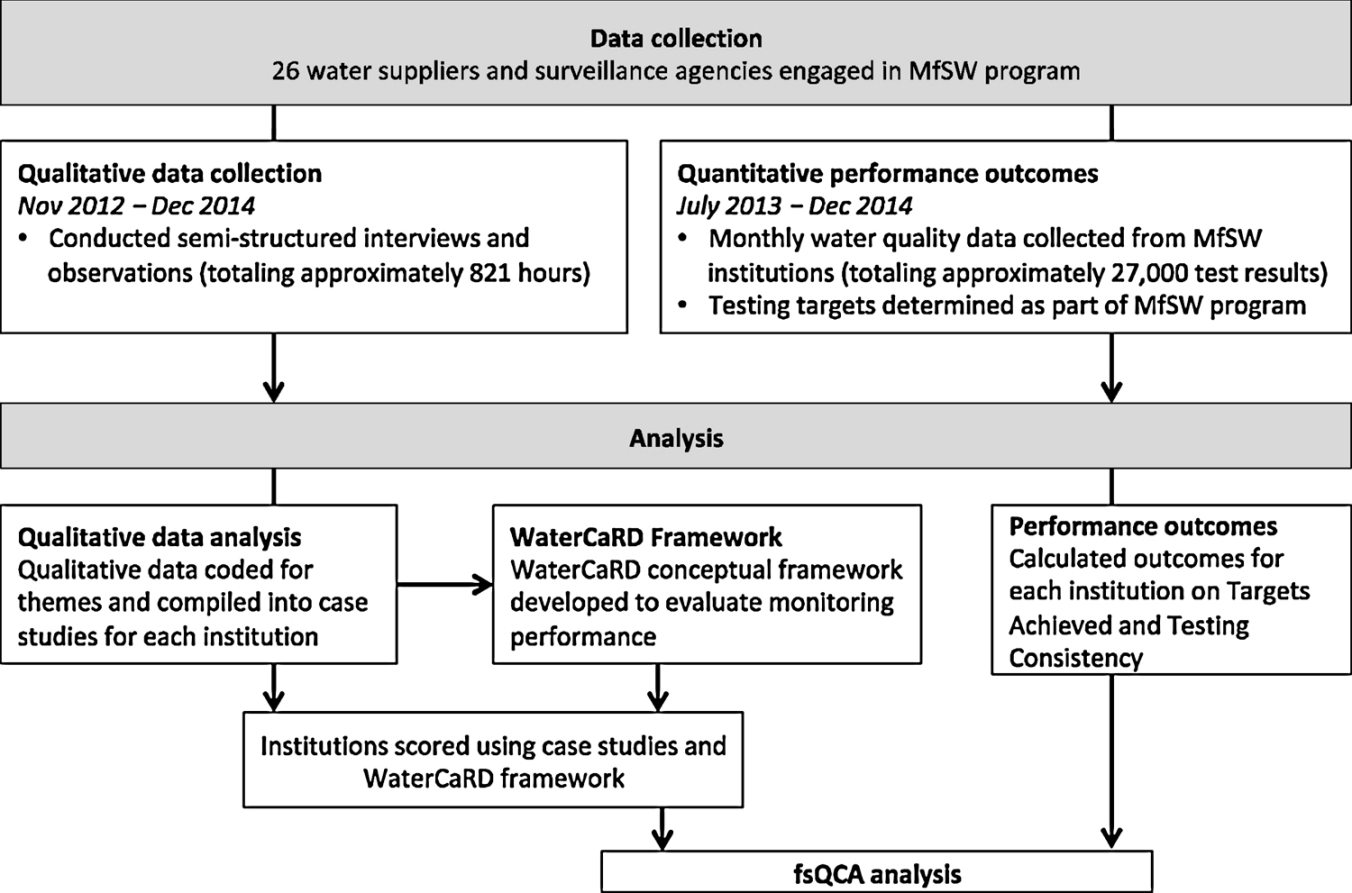
Data collection flow. This figure describes the different steps in data collection and data analysis. The two performance outcomes include (1) Targets Achieved, the overall percent of testing targets met throughout the program duration and (2) Testing Consistency, the percentage of months during which testing targets were met. MfSW = Monitoring for Safe Water; WaterCaRD = Water Capacity Rating Diagnostic; fsQCA = fuzzy-set Qualitative Comparative Analysis.

We employed a comparative case study design, using multiple steps of data collection and analysis (Fig. 1). Using the 26 MfSW water suppliers and surveillance agencies as cases, we conducted qualitative semi-structured interviews and observations, which were summarized into case studies for each institution. Based on our qualitative data and previous analyses of regulated monitoring in Africa (Peletz et al., 2016), we created the Water Capacity Rating Diagnostic (WaterCaRD) to establish a conceptual framework for evaluating the institutional conditions that potentially influenced water quality testing performance. Subsequently, we scored the case studies against the institutional conditions specified in WaterCaRD. In addition, we calculated performance outcomes for each institution by comparing their monthly frequencies for microbial water quality testing with their monthly targets. Finally, we applied fuzzy-set Qualitative Comparative Analysis (fsQCA) to compare institutional WaterCaRD scores with testing performance.

### 2.3 Sampling

Our cases were the 26 African water suppliers and surveillance agencies that participated in this MfSW study. MfSW study participation was determined through an application process, which included submission of an institutional summary and water quality testing records. Our case selection was non-random and prioritized heterogeneity for case study comparisons (Yin, 2009; Berg-Schlosser and De Meur, 2008); details of our institutional selection process are described elsewhere (Peletz et al., 2016).

The MfSW study collaborating institutions encompassed a diversity of institutional structures, sizes, geographical locations, and testing levels across six African countries: Ethiopia, Guinea, Kenya, Senegal, Uganda, and Zambia (Table 1). They included 11 water supply organizations (two national, two provincial, two city/town, and one association of private piped network operators) and 15 surveillance agencies (one national health ministry, three regional laboratories, and 11 district health or water offices) (Table 1). These institutions collectively covered 118 urban water supply networks and 343 public health districts, which included drinking water provision to over 40 million people (Table 1). All of the suppliers served urban populations, and most of the surveillance agencies (12/15) operated in rural settings (Table 1). The formal MfSW collaborations for this study commenced between May-November 2013 and ended in June 2015. Among water suppliers, interview respondents were primarily laboratory technicians, water quality managers, and managing directors; among surveillance agencies, respondents were primarily public/district health officers, environmental health technicians, and community health workers.

### 2.4 Quantitative data collection: institutional testing performance outcomes

From July 2013 to December 2014, MfSW collaborating institutions submitted their microbial water quality test results to Aquaya on a monthly basis via electronic mail, providing approximately 27,000 measurements of fecal contamination (Kumpel et al., 2016). To quantify their microbial testing performance, we defined two performance outcomes: (1) *proportion of testing targets achieved* = total tests conducted / total number of tests required to achieve testing targets since testing commenced (Targets Achieved); and (2) *proportion of months targets were achieved* = months meeting 100% of targets / total months since formal collaborating agreements signed (Testing Consistency). The first outcome metric captured total testing activities, and the second outcome metric captured the consistency of monitoring. Institutions could score >100% for Targets Achieved (i.e., total test numbers exceeded total targets), though 100% was the maximum score for Testing Consistency.

### 2.5 Qualitative data collection and management

To collect information on institutional characteristics, we conducted qualitative semi-structured interviews and observations between November 2012 and December 2014, totaling approximately 821 h (Table 2). The qualitative data collection included in-person needs assessments (229 h); mid-term program assessments (492 h); other meetings including phone calls, country-level focus groups and in-person meetings with institutions and regulators/ministries (100 h), and review of 94 documents (written applications, needs assessment reports, midterm assessment reports, and training reports). We have included our interview guides and observation forms as supplementary information (Supplemental Interview Guides). These multiple sources of qualitative data were useful for triangulating findings. We collected data in English or French (subsequently translated to English), took detailed notes, and also recorded interviews, which were partially transcribed to corroborate notes and document illustrative quotes verbatim. We entered all qualitative data into the NVivo qualitative data analysis computer program (QSR International Pty Ltd. Version 10, 2014) to code for themes; our coding process and list of codes are provided in Table S1. We applied a combined inductive and deductive approach to formulate hypothesized constraints to water quality monitoring, employing the iterative cycle of compiling, disassembling, reassembling, interpreting, and concluding information (Yin, 2011, 2009). We synthesized our qualitative findings into detailed 10-page case studies for each institution, following a standard outline to capture institutional characteristics (example provided as Supplementary Case Study). At this stage, we also communicated with the MfSW collaborating institutions to clarify remaining questions regarding their structures and capacities.

**Table 2:** Time spent on qualitative data collection by country.

Countries	Number of Institutions		Time Spent on Interviews (Hours)			
	Supplier	Surveillance	Needs Assessment	Midterm Assessment	Other meetings^a^	Total
Ethiopia	2	2	24	48	3	75
Guinea	1	0	40	76	3	119
Kenya	4	3	39	87.5	50.5	177
Senegal	0	1	22	33.5	5	60.5
Uganda	2	5	57	160	21.5	238.5
Zambia	2	4	47	87	17	151
Summary	11	15	229	492	100	821

a Other meetings included phone calls, country-level focus group discussions, and in-person interviews (with institutions and regulators or ministries).

### 2.6 Conceptual framework: Water Capacity Rating Diagnostic (WaterCaRD)

We created the Water Capacity Rating Diagnostic (WaterCaRD) to establish a conceptual framework for evaluating the conditions that potentially influenced water quality testing performance. Based on our qualitative data and previous analyses of regulated monitoring in Africa (Peletz et al., 2016), we selected 27 conditions that fell into five categories (Table 3):

1) *accountability*, which examined the extent to which institutions were responsible for achieving national standards and for reporting to consumers and authorities (including direct line ministries);
2) *staffing,* which assessed whether water quality staff were sufficiently skilled and whether staff turnover was effectively managed;
3) *program structure*, which measured the extent to which procedures were in place for monitoring activities such as selecting sampling locations, transporting samples, coordinating testing, and managing data;
4) *finances*, which evaluated the availability of financial resources and the procedures for budgeting and accounting; and
5) *equipment & services*, which determined whether institutions were able to access, procure, and maintain adequate equipment and facilities for testing.

**Table 3:** Institutional conditions assessed by the Water Capacity Rating Diagnostic (WaterCaRD).

Categories	Conditions	Definition / description	Included in fsQCA^a^
Accountability	National standards	Are comprehensive national standards established?	N (no association with outcomes)
	Regulatory reporting	Does the institution submit water quality data to regulatory authorities on a regular basis?	N (no association with outcomes)
	Consumer reporting	Does the institution consistently share test results with consumers?	N (no association with outcomes)
	Enforcement	Do regulatory authorities provide feedback and incentives/penalties for testing?	Y
Staffing	Leadership	Does the institution’s leadership prioritize water quality monitoring?	Y^b^
	Staff roles	Are roles and responsibilities well-defined?	N (insufficient variation)
	Knowledge	Does staff have practical experience and theoretical knowledge of water testing?	Y
	Training	Are training procedures and resources established?	N (no association with outcomes)
	Motivation	Does staff understand the importance of water quality monitoring and internalize this responsibility?	Y^b^
	Staff stability	Does the institution have high staff turnover?	Y^c^
	Staff recruitment	Does the institution have procedures in place for recruiting and training new staff?	Y^c^
	Risk management	Has the institution identified and managed potential risks that may interrupt monitoring?	N (unavailable data)
Program Structure	Methods	Can the institution perform the testing methods correctly?	N (represented by *Knowledge*)
	Use of testing results	Do the testing results give sufficient information for managing water safety and for reporting to authorities?	N (unavailable data)
	Sampling plans	Has the institution developed a sampling plan, considering the local context and national standards?	N (unavailable data)
	Sample collection	Is the institution able to collect a sufficient number of samples on time?	N (unavailable data)
	Transport	Does the institution have some form of transportation to consistently collect samples?	Y
	Quality control	Does the institution consistently conduct quality control procedures?	N (represented by *Knowledge*)
	Data management	Is water quality testing data available and organized in digital files?	N (insufficient variation)
	Remedial actions	Does the institution consistently respond to contaminated tests?	N (no association with outcomes)
Finances	Financial resources	If funding consistent and sufficient to support regulatory requirements for monitoring?	N (no association with outcomes)
	Budgeting	Is there a specific budget for water quality monitoring?	N (no association with outcomes)
	Accounting	Are accounting systems in place to ensure that payments are accurate and timely?	N (unavailable data)
Equipment and services	Equipment	Does the institution have access to distributors with an adequate equipment range?	Y
	Maintenance	Does the institution maintain equipment and track consumable supplies?	N (unavailable data)
	Procurement	Are the procedures for obtaining equipment optimized?	Y
	Infrastructure	Does the institution have a dedicated space for water quality testing with the needed facilities?	Y

a The reasons and process for inclusion in fsQCA is further described in Fig. 2.

b *Motivation* and *Leadership* were combined to create one condition, *Motivation & leadership*.

c *Staff stability* and *Staff recruitment* were combined to create one condition, *Staff retention*.

These five categories are consistent with other assessment frameworks in the water and sanitation sector, though none of the following examples specifically address requirements for water quality testing (Schweitzer et al., 2014). USAID’s Sustainability Index Tool and the Dutch WASH Alliance’s Sustainability Monitoring Framework measure water and sanitation program sustainability using five similar categories of conditions: 1) *environmental,* 2) *social*, 3) *financial,* 4) *technical,* and 5) *institutional*, though *social* is replaced with *management* in the USAID Tool (Schweitzer et al., 2014; AguaConsult, 2015). The Skat Foundation’s Technology Applicability Framework uses the same five categories as the Dutch WASH Alliance (renaming *financial* as *economic*), plus an additional *knowledge* category (Schweitzer et al., 2014). More broadly, institutional capacity building studies have identified similar constructs (Grindle and Hilderbrand, 1995), such as *structures, systems and roles* (i.e., accountability and institutional processes)*; staff and infrastructure* (e.g., staff retention, leadership, and infrastructure)*; skills* (of staff); and *tools* (e.g., finances and equipment*)* (Potter and Brough, 2004).

### 2.7 Qualitative comparative analysis

We applied QCA to identify the conditions that influence monitoring performance in institutions with regulatory mandates to test water quality. Our QCA analysis employed the MfSW collaborating institutions as cases. We established Targets Achieved (outcome 1) and Testing Consistency (outcome 2) as monitoring performance measures, and we applied the WaterCaRD framework to score the MfSW institutions against relevant conditions.

We established numeric values for the outcomes and conditions associated with each case (Fig. 1). To evaluate relationships between outcomes and conditions, we employed the fuzzy set variant of QCA (fsQCA), which specifies that values for the outcomes and conditions range from 0 to 1 on a continuous scale: i.e., outcomes and conditions are scored as values between 0 and 1, with 0 representing complete absence of the outcome or condition and 1 representing complete presence of the outcome or condition; the alternative method of crisp set QCA requires dichotomous outcomes and conditions of either 0 or 1 (Rihoux and Ragin, 2008). Based on our qualitative knowledge and experience with the MfSW program, we calibrated the outcomes using 1 (or 100%) as the full membership threshold, 0 (0%) as full non-membership threshold, and cross-over points of 0.7 (70%) for Targets Achieved (outcome 1) and 0.5 (50%) for Testing Consistency (outcome 2) (Ragin, 2008). To establish numeric values for the conditions, we developed a scoring guide for WaterCaRD by defining a 4-category scale for each of the 27 conditions (Table S2). The scale ranged from a score of 0 for the lowest ranking (i.e., ‘full non-membership’ in QCA terminology) to a score of 1 for the highest ranking (i.e., ‘full membership’ in QCA terminology), and intermediate scores of 0.33 and 0.67 for partial non-membership and partial membership, respectively (Chatterley et al., 2014). We designated 0.5 as the scale midpoint (i.e., ‘cross-over threshold’ in QCA terminology).

Two independent evaluators who were not involved in data collection, framework development, or testing performance analysis scored each of the 26 MfSW collaborating institutions against all 27 conditions in the WaterCaRD framework using the 10-page case studies that summarized the qualitative findings for each institution (an example case study is included in our supplementary material). The first author (RP) then reviewed scores, rescored in the case of discrepancies, and refined the scoring guide to finalize the dataset (i.e., ‘inter-calibrator reliability checks,’ Tables S2 and S3) (Schneider and Wagemann, 2010; Rihoux and Ragin, 2008). Additionally, we summed the 27 WaterCaRD scores for each institution to create a combined capacity score (as a proportion of total available scores, excluding missing data), which we examined for associations with the testing performance outcomes using simple linear regression (Fig. 2).

**Fig. 2:**
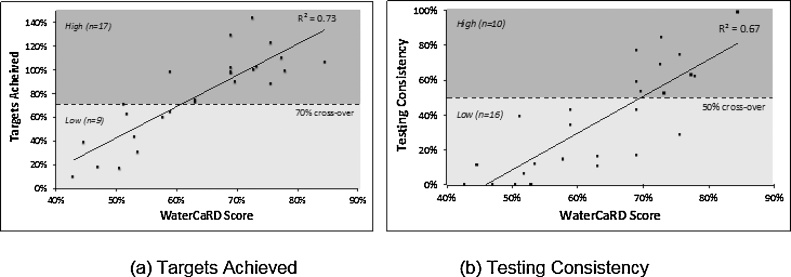
Scatterplots of WaterCaRD scores versus water quality monitoring performance. Each dot represents a testing institution. The WaterCaRD score encompasses ratings on the institutional conditions selected for the QCA analysis as a proportion of total available scores. We measured monitoring performance using two indicators: (a) Targets Achieved (outcome 1), and (b) Testing Consistency (outcome 2). These outcomes were calculated as (a) Targets Achieved = total tests conducted / total number of tests required to achieve testing targets since testing commenced; and (b) Testing Consistency *=* months meeting 100% of targets / total months since formal collaborating agreements signed. We differentiated performance levels into two categories based on the cross-over point (dashed lines), determine by our qualitative knowledge and experience of the MfSW program. For Targets Achieved, 17 institutions were considered high performers (≥70%, the cross-over point); the remaining nine were classified as low performers. For Testing Consistency, 10 institutions were considered high performers (≥50%, the cross-over point); the remaining 16 were classified as low-performers.

**Fig. 3:**
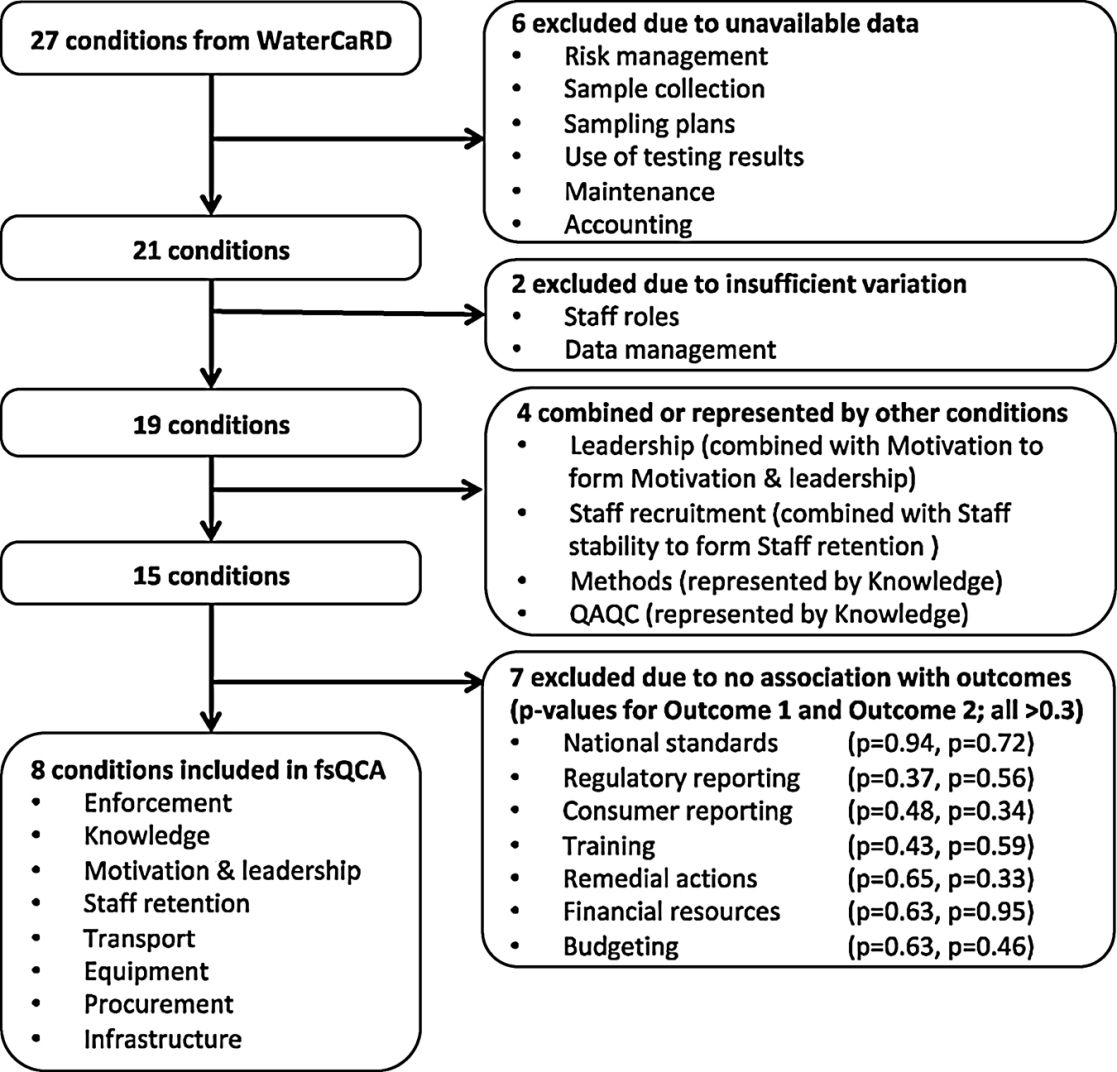
Selection of institutional conditions for fsQCA. Of the 27 institutional parameters, or conditions, that comprise the WaterCaRD scoring framework, we excluded six conditions due to unavailable of data and two due to insufficient variation; four conditions were combined or better represented by other conditions. Finally, we excluded seven conditions due to a low association with the outcomes (Spearman’s correlation coefficient r_s_<0.2, p > 0.3); these p-values are presented for Targets Achieved (outcome 1) and Testing Consistency (outcome 2).

Because QCA requires a limited number of conditions (Schneider and Wagemann, 2010; Berg-schlosser et al., 2008), we reduced our initial 27 WaterCaRD conditions to eight conditions (Fig. 3). Six conditions had poor data availability and two had insufficient variation (<30% variation, ‘domain’ conditions in QCA terminology) (Fig. 3). We also grouped two sets of two conditions: *leadership* merged with *motivation* to be *motivation & leadership*, and *staff recruitment* merged with *staff stability* to form *staff retention.* We eliminated two additional conditions that were a subset of *knowledge*: institutional staff abilities to perform analytical methods (*methods)* and quality control tests (*quality control)* (Fig. 3). Of the remaining 15 conditions, we excluded seven because of a lack of association with the outcome metrics based on our qualitative data, bivariate plots, and Spearman’s coefficients (for both outcomes, r_s_<0.2, p > 0.3) (Chatterley et al., 2014). Ultimately, our QCA examined the influences of eight conditions on testing performance: 1) *enforcement,* 2) *knowledge,* 3) *motivation & leadership,* 4) *staff retention,* 5) *transport,* 6) *equipment,* 7) *procurement,* and 8) *infrastructure* (Fig. 3, Table 3).

We summarized the values or scores for the outcomes and conditions in a data table (Table S3) and then used the fsQCA software package (Ragin, Charles, and Sean Davey. 2014. fs/QCA [Windows], Version [2.5]. Irvine, CA: University of California) to translate information from the data table into a “truth table” that contains all observed configurations of conditions and outcomes (Ragin et al., 2008; Rihoux and Ragin, 2008). In fsQCA, this truth table is minimized using fuzzy logic: i.e., two cases producing the same outcome but differing by only one condition are simplified to omit the redundant condition. This iterative process compares conditions across all cases to eliminate redundant conditions, which results in the identification of the causal conditions (or combinations of conditions, termed ‘solutions’ or ‘pathways’) that are *necessary* or *sufficient* to explain the outcome of interest. *Necessary* conditions are required to achieve the outcome of interest (i.e., they are highly shared among pathways that lead to the desired outcome), though they may not be adequate, on their own, to produce the outcome. *Sufficient* conditions are found in some, but not all, pathways or solutions to achieve the outcome of interest. The pathways are the most logically succinct combinations of necessary and sufficient conditions to produce the outcome of interest (Rihoux and Ragin, 2008; Jordan et al., 2011).

Two “goodness-of-fit” measurements are used to assess QCA outputs: *consistency* and *coverage*. *Consistency* measures the proportion of cases with the causal condition(s) that exhibit the outcome of interest. Conditions with a consistency score of at least 0.9 are considered necessary (i.e., the majority of cases with the condition(s) will present the outcome, though this is not directly 90% of cases because of the potential for partial membership and partial non-membership in fsQCA) (Rihoux and Ragin, 2008). *Coverage* measures the proportion of cases exhibiting the outcome that can be explained by the condition(s) or pathways presented; high coverage scores indicate that the given pathways represent many of the studied cases (Rihoux and Ragin, 2008). All pathways use a sufficiency consistency threshold of 0.80 and present the intermediate solution, which assumes a positive influence of each condition on the outcome based on institutional knowledge (Ragin et al., 2008).

### 2.8 Ethical review

The Western Institutional Review Board (WIRB) (Olympia, WA, USA) determined that this study was exempt from full ethical review under 45 CFR 46.101(b)(2) of the Federal Common Rule in the USA. To protect confidentiality, we have not specified the names of institutions or individuals; in data tables and figures, we labeled institutions using the first letter of the country where they are located and a number.

## 3 Results

### 3.1 Institutional monitoring performance and WaterCaRD scores

**Fig. 4:**
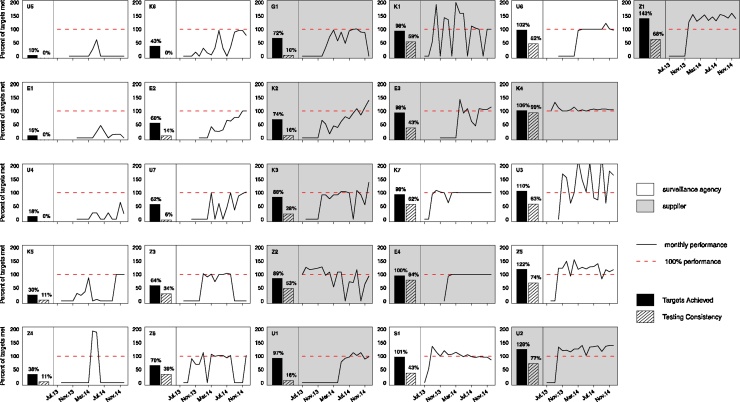
Graphical displays of water quality monitoring performance. Each graph depicts the monthly monitoring levels achieved by a MfSW collaborating institution between when they entered the MfSW program (July 2013 earliest) and December 2014. Months with no data were prior to the institution entering the MfSW program. Performance levels, shown as a solid black line, were calculated as the percentage of microbial testing targets met monthly. Testing targets and program start dates differed among institutions. We also indicate two aggregate performance metrics: Targets Achieved, the overall percent of targets met throughout the program duration (outcome 1, solid bar) and Testing Consistency, the percentage of months during which targets were met (outcome 2, striped bar). Surveillance agencies are indicated with a white background, and piped water suppliers are indicated with a gray background. The graphs are ordered vertically according to monitoring performance as measured by Targets Achieved (solid bar). The lowest performer is placed in the top left position, and the highest performer is in the top right position.

Among the 26 regulated monitoring institutions, or cases, microbial water quality testing performance, as measured by Targets Achieved (outcome 1), ranged from 10% to 143%, with a median of 89% [interquartile range (IQR): 60%–101%] (Fig. 4). Seventeen institutions met at least 70% of their testing targets (the cross-over point) and were therefore classified as high performers in terms of Targets Achieved (Fig. 2a). Of the remaining nine institutions (classified as low performers), all were surveillance agencies and eight were considered rural (Fig. 4).

Microbial water quality testing performance, as measured by Testing Consistency (outcome 2), was generally lower because this measure penalized institutions that fluctuated over time in their monitoring performance or experienced long lag-times before they started MfSW-supported monitoring activities. Institutional performance based on Testing Consistency ranged from 0% to 99%, with a median of 37% [IQR: 11%–62%] (Fig. 4). Ten institutions met their testing targets for at least 50% of months (the cross-over point) and were therefore classified as high performers in terms of Testing Consistency (Fig. 2b). Of the remaining 16 institutions (classified as low performers), 11 were surveillance agencies and nine were considered rural (Fig. 4). We provide additional data for microbial water quality testing performance in Table S3.

Combined WaterCaRD scores for the 26 cases ranged from 43 to 85%, with a median of 66% [IQR: 53%–73%]. The combined scores were significantly associated with the performance levels for both outcomes (p < 0.001; Targets Achieved R^2^ = 0.73, Testing Consistency R^2^ = 0.67) (Fig. 2).

### 3.2 Necessary and sufficient conditions

**Fig. 5:**
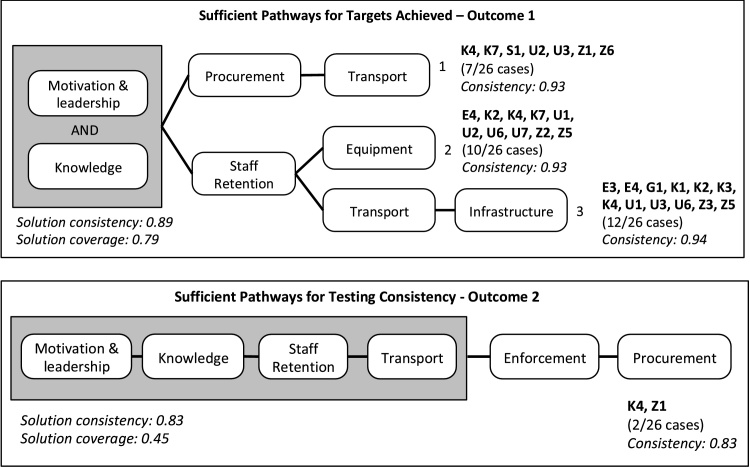
Pathways or combinations of conditions for that promote high monitoring performance, as identified using fsQCA. Monitoring performance was measured using two metrics: Targets Achieved - Outcome 1; and Testing Consistency - Outcome 2. The pathways present the necessary conditions in shaded boxes. The cases in each pathway are listed in bold to the right of each pathway. *Consistency* measures the proportion of cases with the condition(s) that exhibit the outcome of interest. *Coverage* measures the proportion of cases exhibiting the outcome that can be explained by the condition(s) or pathways presented. Unique coverage was 7% for the first two pathways and 8% for the third pathway; nine cases were in multiple pathways.

fsQCA identified all eight of our selected conditions as necessary or sufficient for achieving high levels of microbial water quality testing performance, as measured by Targets Achieved (outcome 1) and Testing Consistency (outcome 2) (Fig. 5). These eight conditions are summarized below and additional detail is provided in Table 4.

**Table 4:** Additional information on conditions for each type of institutions.

Condition	Water Suppliers	Surveillance agencies	Both
Enforcement	• In Zambia and Kenya, suppliers regularly reported to regulators and were periodically subject to audits. Regulators rated suppliers on their overall performance, including testing. • Other countries had little to no enforcement for testing. Some suppliers did not send testing data to any external regulatory bodies.	• There was a lack of enforcement; though water testing data was often sent to relevant national ministries (e.g., Ministry of Health), institutions received minimal or no feedback.	• Except for the suppliers in Kenya and Zambia, there was a substantial lack of enforcement for water testing. Most institutions were not penalized or incentivized for testing.
Knowledge	• Generally, suppliers only had a few staff dedicated to water quality, which enabled them to specialize and build expertise in water testing. • Most staff had theoretical training in water testing via degrees, diplomas or certificates on water-related courses.	• Often multiple staff were involved in testing, who were also responsible for other activities. • Most staff did not have theoretical training in water testing via degrees, diplomas, or certificates; staff had difficulty building substantial theoretical knowledge in water testing through short training courses.	• Lack of experience or knowledge led to testing methods often being performed incorrectly. • Staff with knowledge and experience in water testing often led testing activities and informally mentored other staff.
Motivation & leadership	• Consistent monitoring resulted in job ownership for dedicated staff. • The procurement of high quality equipment motivated staff. • Effective leaders ensured that sufficient resources were available for testing.	• Most surveillance staff received incentive payments to cover transportation and lunch allowances during sample collection and testing. • Staff that did not receive incentive payments often prioritized other incentivized activities (e.g., polio campaigns) over water quality monitoring.	• Training opportunities and the involvement of external partners motivated staff. • Attentive supervision encouraged staff to work productively. • Effective leaders managed the procurement and distribution of consumables and supplies.
Staff Retention	• Because often only a few staff managed testing activities, testing programs were sometimes interrupted while staff left (temporarily or permanently).	• Internal staff transfers were a common challenge (e.g., district health staff moving to other districts). • Some trained all their staff on water testing to minimize disruptions from internal staff transfers. • Qualified staff was difficult to retain in rural areas.	• Staff turnover was a challenge if new staff did not have adequate knowledge and experience. • Changes in government structures, such as the decentralization of national to county governments in Kenya, resulted in staff transitions.
Transport	• Most suppliers had vehicles or motorbikes for sample collection, but these were not always available (e.g., used by another department or didn't have fuel).	• Most used motorbikes, public transport and/or walked for sampling. If vehicles/motorbikes were available, they were shared with other departments or activities. • Some had to cover vast geographical areas resulting in high transportation costs. • Sometimes motorbikes were broken down and in need of spare parts for repair.	• Poor road infrastructure, particularly in mountainous areas and during the rainy season, made it difficult to collect and transport samples.
Equipment	• Suppliers often had previous experience with testing and therefore were somewhat familiar with equipment providers.	• Some surveillance agencies had minimal testing experience and therefore lacked contact information for equipment providers.	• Some institutions had easy access to distributors. • Most equipment/supplies were imported, which increased equipment cost and delivery time. • Occasionally, distributors ran out of stock.
Procurement			• Multi-step procurement processes delayed testing, particularly in larger institutions, though the processes aimed to minimize corruption. • Multiple bids were often required, though sometimes there was only one equipment provider in the country. • Miscommunication resulted in some institutions receiving the incorrect equipment.
Infrastructure	• Many had established laboratories for testing, some of which were ISO certified. • Water suppliers used electricity for operating their treatment plants and distributing water supplies. Therefore, power outages interrupted water distribution, which prevented the collection of water samples from the distribution network.	• Most lacked established laboratories; testing was often conducted in an office, school, or hospital. • Simpler testing techniques (e.g., presence/absence) were more common and had less infrastructure requirements. • If laboratories existed, they were sometimes used for other medical tests.	• External donors sometimes funded laboratory infrastructure. • Power outages interfered with the refrigeration of media and incubation of samples.

#### 3.2.1 Knowledge

Most institutions had knowledge and experience in water testing (92%, 24/26, scored ≥0.67 on the fsQCA scale, which ranged from 0 to 1). “*Those that are in charge of water quality… I am confident* [in their abilities]” (Quality Assurance Officer, Z2). However, differences in staffing structures between water suppliers and surveillance agencies appeared to influence testing knowledge and experience. Water suppliers generally maintained a few staff members with dedicated responsibilities for water testing, which promoted specialization and the development of expertise. In comparison, testing staff in surveillance agencies was often responsible for other unrelated activities. “[They] *are not specialized in water testing but … they take turns doing it*” (Regional Director, S1). In addition, surveillance staff usually had not received formal training (e.g., a degree, diploma or certificate) in the theoretical aspects of water monitoring. “*The health assistants, they get the knowledge from the field*” (Health Inspector, U6). Surveillance staff did often attend short training courses on water quality testing, but these practical exercises rarely provided the background knowledge required for understanding testing methods and for designing testing programs. *“The practical exercise was made only once in that training and colleagues did not grasp it very well”* (District Health Officer, U6*).*

#### 3.2.2 Motivation & leadership

Most institutions had motivated staff with effective leadership that prioritized water quality monitoring (77%, 20/26 scored ≥0.67). *“At the highest level, from top to bottom, there is full commitment of the staff. We are committed just because water quality monitoring is one of our missions”* (Regional Manager, S1). Attentive supervision by leadership encouraged the staff to work productively and thoroughly: “[I] *visit the water lab there to see that somebody is doing something…. I* [am] *strict in terms of supervision of the exercise, so these guys are serious and they can go pick some data*” (Acting Managing Director, K3). In addition, some institutions used the incentive payments to promote staff motivation in some institutions, “*Community health workers play a very important role and we included some incentives for them in our initial budget*” (Public Health Officer, K7).

Of the six lowest scoring institutions for Motivation & Leadership (scores of ≤0.33), four were district health offices; four were also classified as rural. The staff at these institutions was generally responsible for a broad range of public health services (e.g., vaccination programs and restaurant food inspections) that were often prioritized above water quality monitoring activities. *“I think we have not given a sufficient time to do this* [testing] *and then to couple with that we have not oriented everybody on this”* (District Health Inspector, U5). District health offices often had inadequate staffing to cover all of their activities, which led to lower institutional priorities for water quality monitoring, even if individual staff were motivated to conduct testing.

#### 3.2.3 Staff retention

Among 69% (18/26) of institutions, staff retention was managed effectively, due to low staff turnover and/or recruitment of new staff that were adequately trained (fsQCA score ≥0.67). “*When they are transferring* [a] *quality control technician, they replace with a quality control technician and all* [of] *our quality control technicians are trained*” (Quality Control Technician, U1).

Nevertheless, staff replacement created tangible challenges when new personnel lacked the required skills to conduct testing. “*The one who was trained for monitoring, sampling and testing was redeployed to hold another position and someone else has been* [hired]… *who was not trained on the same, water quality monitoring, sampling and testing. That became a very big challenge for me… I need to start afresh training the person*” (Quality Assurance Officer, Z2). Surveillance agencies often faced challenges retaining staff in rural areas. “*For the staff that may be brought* [up] *in town, grown in town, educated in town…* [and then] *brought* [to] *a rural place like this one, it becomes more of a challenge…. because their minds is not focused on staying*” (Public Health Officer, Z6). Temporary leave sometimes caused problems for water suppliers with only one or two staff responsible for water testing. “*The person who is supposed to be doing that is not around, now that he is on leave and none of us can use a motorbike”* (Water Quality Manager, K2).

#### 3.2.4 Transport

Most institutions (23/26, 88%) generally had transportation resources for sample collection (score ≥0.67), though many faced occasional challenges. Only 23% (6/26) of institutions had dedicated transport for sample transportation (score = 1); other institutions had to coordinate vehicle use between multiple activities or departments. “*We have many other activities to handle at times… So we design a monthly plan* [for]*… the cars…. I try to organize shifts for teams to go and do sampling*” (Regional Director, S1).” In some cases, available vehicles or motorbikes were broken down and could not be repaired due to a lack of spare parts or funds. “*Motorbikes are there* [but]… *repair and servicing… is where challenges are, and again it comes back to the spare parts… In fact as we are speaking right now we have got quite a number of them* [motorbikes] *that are parked* [i.e., broken down]” (Environmental Health Technologist, Z6). Additionally, transporting samples was difficult in areas without paved roads that became muddy during the rainy season. “*When it rains, there* [are] *some areas where we pick samples and* [it] *is difficult to access; the motorcycle cannot reach because it is slippery, so you have* [to] *walk*” (Area Manager, U2).

#### 3.2.5 Equipment

More than half, 54% (14/26), of institutions faced challenges in acquiring the equipment needed for water testing (score ≤0.33). Because most equipment was imported, institutions had to either make purchases directly from international providers or place orders with local distributors. Both options resulted in long delivery times. “*The challenge really in Zambia is that most of the items are not manufactured locally… So the lead time will vary from two weeks to four weeks, sometimes even six to eight weeks*” (Acting Managing Director, Z1). Equipment distributors sometimes ran out of stock, interrupting institutional testing activities. “*We conduct testing… except when we don't have media. … Sometimes, they* [i.e., distributors] *can run out, and when they run out, it impacts our activities*” (Water Quality Director, G1). Additionally, some institutions without prior testing experience were unsure on how to contact equipment providers. “*I do not have any contacts of those kind of people dealing with steam sterilizers and so on*” (District Health Inspector, U5).

#### 3.2.6 Procurement

Among 73% (19/26) of institutions, the official procurement procedures were tedious and inefficient (score ≤0.33). Procurement regulations often specified complex multi-step procedures for purchasing equipment to minimize corruption, particularly within larger institutions. “*The procurement regulation, that’s* [what] *makes procurement very, very slow… First anything to be procured should be in the plan or in the budget of the district…* [The] *approval of* [the] *budget heavily involve*[s] *the politicians, district politicians from the various committees and these committees do not meet* [often], [only] *once in two months*” (Public Health Officer, U7). Additionally, sometimes the procurement process required multiple bids, even when multiple equipment providers were not available in the country. “*The finance regulation can’t allow us to purchase unless three suppliers provide us with pro forma… we are waiting patiently what is going to come next*” (Laboratory Staff, E1).

#### 3.2.7 Infrastructure

Fifty-four percent (14/26) of institutions had a dedicated testing space that usually had a functioning electrical connection (score ≥0.67). Many of the water suppliers had laboratories that they could use for testing (8/11 suppliers scored ≥0.67). Most of the health surveillance institutions lacked established laboratories (9/15 scored ≤0.33); staff conducted testing in a common location such as an office, school, or hospital. “*We need a good infrastructure, we are struggling to get that*” (Managing Director, E2). However, surveillance agencies were also more likely to use simpler microbial testing techniques such as presence/absence testing that did not require laboratory facilities. For almost all institutions, power outages interfered with refrigerating media, incubating samples, and/or collecting samples from piped systems (since water distribution was often dependent on electricity). “*The challenge we have … is intermittent power supply, … the ideal situation is… after sampling … we incubate* [samples] *for* 18 h*, 15 to 18, but here is the situation, within maybe* 8 h *the power has gone, so it is a big challenge*” (Quality Control Technician, U4).

#### 3.2.8 Enforcement

We found that most institutions (77%, 20/26) did not receive any feedback, incentives, or penalties from regulatory authorities for testing activities (score ≤0.33), despite submitting testing data to relevant government ministries. With respect to surveillance agencies, we defined regulatory authorities as relevant line ministries. For example, national Ministries of Health often oversee district health offices. “*That report is sent to the ministry…* [but it is] *very rare for the Ministry to* [give] *feedback unless during* [an] *outbreak*” (Environmental Health Officer, Z5). Some institutions did not send data to any regulatory authorities because *“they have not asked us to submit* [data]” (District Health Inspector, U5).

Enforcement was strongest in Zambia and Kenya (six suppliers scored ≥0.67), where an independent regulator provided oversight and feedback to suppliers. *“All the water utilities companies are regulated…* [the regulators] *send their inspectors to come and verify the information that we sent. They don't only verify the numbers, but they also get into details about sampling: the way the method was conducted* [and] *if we are following the checklist”* (Technical Director, Z1). Regulators rated supplier performance on a number of activities, including water testing; these performance ratings were publicly available and were “*meant to stimulate action, so hence making service provision better”* (Managing Director, K1).

### 3.3 Pathways to successful testing: fsQCA results

#### 3.3.1 Necessity

According to fsQCA of the 26 institutional cases, two conditions, 1) *knowledge* and 2) *motivation & leadership,* were necessary conditions for high monitoring performance, as measured by Targets Achieved (outcome 1) (Fig. 5, Table S4). Among the 17 cases classified as high performers in terms of Targets Achieved (i.e., outcome 1 ≥ 70%, the cross-over point), all but two institutions scored ≥0.67 for these two conditions; the exceptions scored 0.33 for *motivation & leadership* and had outcomes near the cross-over point (70% for Z6 and 74% for K2) (Table S3). In the case of Testing Consistency (outcome 2), four conditions were necessary: 1) *knowledge*, 2) *motivation & leadership*, 3) *staff retention* and 4) *transport* (Fig. 5, Table S4). Among the ten cases that exhibited high Testing Consistency (outcome 2 ≥ 50%, the cross-over point), all scored ≥0.67 for these four conditions (Table S3). We did not find any paradoxical results, defined as conditions that are necessary for achieving both the positive and negative outcome (Ragin, 2008), when examining the necessity of each condition for the negation of each outcome.

#### 3.3.2 Pathway analysis

Three distinct pathways led to Targets Achieved (outcome 1), with an overall solution consistency of 0.89 and coverage of 0.79 (Fig. 5); consistency for each pathway exceeded 0.90. It is important to note that coverage and consistency values do not translate directly into proportions of cases because of the potential for partial membership and partial non-membership in fsQCA (Rihoux and Ragin, 2008). Of the 17 cases that had high scores for all causal conditions (≥0.67) in one of the three pathways, 14 exhibited the outcome (outcome 1 ≥ 70%, the cross-over point) (Table S3). In addition, 14/17 of the cases classified as high performers (outcome 1 ≥ 70%) had high scores for all causal conditions (≥0.67) for at least one of the pathways (Table S3). Unique coverage was 7% for the first two pathways and 8% for the third pathway; nine cases were in multiple pathways (Fig. 5).

In addition to the necessary conditions of 1) *knowledge* and 2) *motivation & leadership*, the first pathway included *procurement* and *transport*, representing seven cases (Fig. 5). These seven cases include a range of institutional structures across four countries: one city supplier in Kenya (K4), one regional supplier in Zambia (Z1), one private water operators association in Uganda (U2), two district health offices in Zambia and Kenya (Z6, K7), one regional surveillance agency in Uganda (U3), and one national surveillance agency in Senegal (S1) (Fig. 5). Among these seven cases, staff was able to efficiently navigate *procurement* procedures and organize *transport*: staff either had access to cars/motorbikes or they were provided with a transportation allowance (all seven cases scored ≥0.67 for these two conditions). For example, the water quality manager of the national agency responsible for water quality surveillance in Senegal effectively coordinated the *procurement* of equipment and consumables and *transport* (vehicles and fuel allowances) to ensure that water testing targets were achieved.

The second pathway included *staff retention* and *equipment,* representing ten cases across four countries: two city suppliers in Kenya (K2, K4), two regional suppliers in Ethiopia and Zambia (E4, Z2), one national supplier in Uganda (U1), one private water operators association in Uganda (U2), four district health offices in Kenya, Uganda, and Zambia (K7, U6, U7, Z5) (Fig. 5). All of these ten cases scored ≥0.67 for *staff retention* and *equipment* (Table S3). For example, the national water supplier in Uganda exhibited good *staff retention* (i.e., minimal staff turnover) and had relatively easy access to *equipment* through Ugandan distributors.

The third pathway included *staff retention*, *transport*, and *infrastructure*, representing 12 cases across five countries: four city water suppliers in Kenya (K1, K2, K3, K4), two regional water suppliers in Ethiopia (E3, E4), two national suppliers in Guinea and Uganda (G1, U1), three district health offices in Uganda and Zambia (U6, Z3, Z5), and one regional surveillance agency in Uganda (U3) (Fig. 5). These cases demonstrated adequate *staff retention* (i.e., minimal staff turnover), *transport* (i.e., staff either had access to cars/motorbikes or was provided with a transportation allowance), and *infrastructure* (i.e., established spaces dedicated to water testing). For example, one regional water supplier in Ethiopia (E4) had two *staff* consistently dedicated to water quality testing, vehicles available for sample *transport*, and an established laboratory *infrastructure.*

With respect to Testing Consistency (outcome 2), our solution included all conditions except *infrastructure* and *equipment*, with a consistency of 0.83 and coverage of 0.45, representing two of the 10 cases that demonstrated high performance (outcome 2 ≥ 50%) (Fig. 5) (Table S3). This solution is similar to the first pathway for Targets Achieved (including *motivation & leadership, knowledge, transport,* and *procurement),* with the addition of *staff retention* and *enforcement* (Fig. 5). The two cases were the only cases that had high scores for all causal conditions in the pathway (≥0.67), and they comprised a regional water supplier in Zambia (Z1) and a city water supplier in Kenya (K4) (Table S3). Both suppliers demonstrated strong *motivation & leadership* and *knowledge* of the managing director and other leadership staff, which helped facilitate *staff retention*, vehicle availability for sample *transport*, and efficient processes for *procurement*. Additionally, these suppliers regularly reported to water regulatory authorities that audited and rated their testing activities, as a form of *enforcement*.

## 4 Discussion

### 4.1 Summary of main results

Initiatives to improve water quality monitoring activities in low-resource settings tend to focus on hardware provision (laboratories, diagnostic equipment, data management tools) and staff training. The results of these efforts, however, are rarely measured, and the generally poor performance of regulated testing programs in many developing countries suggests that their long-term impacts are limited. We now show that even after the provision of laboratory equipment, staff trainings, and financial incentives for conducting microbial water quality tests, monitoring performance varied greatly among water suppliers and surveillance agencies across multiple African countries (Fig. 4). These results indicate that a better understanding of the additional conditions that influence monitoring performance is needed to guide effective capacity building.

Based on the application of fsQCA to evaluate the causal conditions that underlie water quality testing abilities, we determined that high institutional capacity in two areas was necessary for achieving strong performance: 1) *motivation & leadership*, defined as committed staff and effective leaders that prioritized water quality monitoring; and 2) *knowledge*, defined as trained staff with dedicated monitoring responsibilities (Fig. 5, Table 4). High capacity scores in both of these areas predicted testing performance, as measured by two indicators: 1) Targets Achieved (outcome 1), and 2) Testing Consistency (outcome 2). High capacity scores for two additional institutional conditions were also necessary for strong testing performance, as measured specifically by Testing Consistency: 1) *staff retention*, defined as levels of staff turnover; and 2) *transport*, defined as the availability of vehicles for sample collection and road conditions (Fig. 5, Table 4).

In addition to the necessary conditions for achieving targeted levels of water quality testing, fsQCA identified multiple combinations, or pathways, of institutional conditions that contributed to monitoring performance as measured by outcome 1 (Fig. 5). These pathways included *procurement* and *transport* (together representing pathway 1); *staff retention* and *equipment* (together representing pathway 2); and *infrastructure* (together with *staff retention* and *transport*, representing pathway 3) (Fig. 5). In the case of outcome 2, *enforcement* and *procurement* together contributed to monitoring achievement by two of the 10 institutions that were classified as high performers (Fig. 5).

All eight of the conditions that we tested were represented among the different pathways that contributed to high monitoring performance and most were found in multiple pathways. However, three of the conditions, *equipment*, *infrastructure*, and *enforcement* were only represented in a single pathway (Fig. 5). Furthermore, *enforcement* was a causal condition for only a few of the institutions that achieved high monitoring performance according to outcome 2 (Fig. 5). As discussed below, the more limited influences of *equipment* and *infrastructure* on monitoring performance may reflect the abilities of motivated water quality managers to address practical constraints such as poor laboratory conditions: for example, by relying on portable testing kits. In the case of *enforcement*, the MfSW program structure included enforcement functions that may have diminished the influence of local regulatory agencies; this issue is further described below.

### 4.2 The importance of individuals

Our identification of personnel attributes, including *motivation & leadership, knowledge,* and *staff retention*, as key conditions that drive institutional monitoring performance reflects the common understanding that employees are an organization’s “greatest asset” (Spitzer-Lehmann, 1990). Similarly, analyses of sanitation programs has found that “natural leaders” led to more successful Community Led Total Sanitation (CLTS) programs (i.e., increased latrine coverage and decreased open defecation) (Crocker et al., 2016), and “local champions” were present in well-managed school sanitation programs (Chatterley et al., 2014, 2013). A study of community-based drinking water organizations also showed that staff expertise and leadership played a key role in organizational performance (Madrigal et al., 2011).

Our results indicate that in weak regulatory environments (i.e., where there are few penalties for not meeting testing targets), monitoring performance is often determined by the priorities of institutional leaders and their abilities to establish and maintain appropriate personnel: committed individuals find solutions to the technical and logistical challenges that are inherent in water testing programs. In contrast, managers who are not motivated to monitor water safety are less likely to maintain the required staff or find support for testing activities.

Though staff *knowledge* was a necessary condition for monitoring performance, our bivariate analysis did not find any association between *training* (defined as structured educational activities) and institutional abilities to achieve testing targets (Fig. 3). This finding suggests that the short training courses on water quality testing that are generally included in capacity building programs have limited utility; rather, staff with appropriate educational backgrounds and dedicated responsibilities for monitoring water safety are more effective for ensuring adequate testing.

In addition, our bivariate analysis did not detect a relationship between *remedial actions* (defined as water safety management triggered by contaminated samples) and testing activity, which appears to contradict the finding that committed water safety managers and staff are critical for monitoring performance (Fig. 3). This contradiction suggests that even in institutions with strong testing programs, there may be limited application of water quality data to guide water safety management. This gap between data collection and the actual use of the resulting information, i.e., where data collection is seen as a “box checking” activity with limited practical significance, is likely a common challenge for water testing programs in settings where there is little capacity to improve water quality.

The strategies employed by committed management and dedicated staff for addressing the technical and logistical challenges of monitoring programs varied, as indicated by the multiple combinations, or pathways, of causal conditions that contributed to strong monitoring performance (Fig. 5). These pathways emphasize the obvious requirements for technical (*equipment* and *infrastructure*) and logistical (*transport*) capacity, and efficient *procurement* processes that facilitate efficient capacity building, as resources become available (Fig. 5).

### 4.3 MfSW influences on monitoring performance

Each of the eight institutional conditions that we selected for fsQCA were either necessary for or played a role in achieving water quality testing goals (Fig. 5). However, we did not include a number of other conditions in our fsQCA because bivariate analysis determined that institutional scores for these conditions were not associated with monitoring outcomes, e.g., *training* and *remedial actions* as discussed in the previous section (Fig. 3). It is likely that the MfSW intervention influenced institutional capacities in some of these conditions, thereby, obscuring their effects on monitoring performance. For example, capacity scores for both *financial resources* and *budgeting* were not associated with performance (Fig. 3). Yet, monitoring programs clearly require sufficient and sustained funding to maintain equipment, transportation, facilities, and salaries (Delaire et al., 2017). The lack of association between financial capacity and performance is probably because the MfSW intervention mitigated funding constraints by providing collaborating institutions with start-up grants and per-test payments.

Similarly, the monthly reporting requirements that the MfSW program imposed on collaborating institutions may have reduced the effects of actual reporting activities (*regulatory reporting* and *consumer reporting*) on performance (Fig. 3). In addition, the requirement for MfSW collaborating institutions to establish program-related targets for microbial water quality monitoring likely diminished the role of *national standards* in determining their monitoring levels and performance (Fig. 3). Our previous research has also shown that in the absence of national standards for testing activities, monitoring institutions in African countries rely on the WHO Guidelines for Drinking Water Quality (Peletz et al., 2016). It is also possible that the poor enforcement of reporting requirements and compliance with water quality standards has diminished their credibility in practice (Jenson, 1967; Bartram, 1996; Okun and Lauria, 1992).

Finally, accountability is recognized as a key driver of performance in the water sector (Andrés et al., 2013; Madrigal et al., 2011). Yet, *enforcement* had a limited role in determining the monitoring performance of the MfSW collaborating institutions: high *enforcement* scores were only identified as a causal condition for a subset of the institutions that were classified as high performers according to outcome 2, Testing Consistency (Fig. 5) (Table S3). Again, the MfSW program may have moderated the influence of *enforcement* on monitoring performance since collaborating institutions were required to submit monthly records of their testing activities. This requirement effectively served as an enforcement mechanism.

### 4.4 The challenge of rural monitoring

Among the 26 MfSW collaborating institutions, most of the weaker performers were surveillance agencies responsible for monitoring drinking water quality in rural areas (Fig. 4). These surveillance agencies were largely represented by District Public Health Offices with mandates for multiple public health activities, ranging from managing vaccination campaigns to conducting food safety inspections. Too many responsibilities, inadequate government support, and a lack of dedicated staff for water safety management made it difficult for most of the District Public Health Offices to achieve their monitoring targets. Furthermore, donor-funded initiatives often determined operational priorities.

Nevertheless, in most countries, public health agencies remain the only institution with formal requirements for testing and ensuring the quality of water supplies that are not managed by a licensed operators (Rahman et al., 2011). The constraints faced by District Public Health Offices suggests that customary efforts to improve their monitoring capacity (i.e., by providing testing kits), will struggle to achieve sustainable improvements in performance. Rather, improved monitoring of rural water supplies may also require institutional innovations such as partnerships with private sector actors who can provide cost-effective testing services.

### 4.5 Study limitations

We selected the 26 MfSW collaborating institutions that served as cases for this study based on applications that they submitted for participation in the MfSW program (Peletz et al., 2016). This non-random selection process is not necessarily a limitation for QCA, which seeks to include the widest range of outcomes (Kaminsky and Jordan, 2017). However, specific attributes of the MfSW program may limit the generalizability of our results. For example, associations between performance and institutional scores for both *financial resources* and *budgeting* were likely diminished by MfSW financial inputs, which included start-up grants and per-test payments. Likewise, MfSW reporting requirements probably reduced associations between performance and scores for both regulatory reporting and consumer reporting. Finally, each MfSW collaborating institution was required to develop a microbial water quality testing plan, which may underlie the lack of association between scores for *national standards* and performance.

We also excluded multiple institutional conditions (*risk management*, *sample collection*, *sampling plans*, *use of test results*, *maintenance*, and *accounting*) from our fsQCA because we were not able to collect sufficient data to accurately score institutional capacity in these areas (Fig. 3). It is probable that capacity levels for some of these excluded conditions influence both monitoring performance and the subsequent use of data to guide water safety management.

Despite the influences of the MfSW program and our inability to evaluate all conditions, we did distinguish critical elements of institutional capacity that contributed to variations in monitoring performance among the MfSW institutions and identified gaps in traditional monitoring improvement efforts. However, notwithstanding our identification of these critical conditions, comprehensive evaluations of institutional capacities in all areas related to water quality management remain important for strengthening monitoring programs.

### 4.6 Conclusions: building water safety capacity

The JMP has now included assessments of drinking water quality in their measurements of progress towards SDG Target 6.1, which specifies universal and equitable access to safe and affordable drinking water for all by 2030 (WHO/UNICEF, 2017). This recognition that water quality data is essential for managing drinking water safety should, optimally, increase both political will and resource allocations for addressing current deficiencies in local data collection and analysis. Better local data, in turn, will help address challenges to safe and affordable service delivery and help guide actions to improve water quality.

Our results indicate, however, that improving local data collection and analysis requires a shift from traditional supply-side interventions (i.e., provision of laboratories, equipment, and training) to a more holistic consideration of the multiple conditions that influence a monitoring program’s success. For example, the critical conditions for strong monitoring performance, particularly in weak regulatory environments, include management that is motivated or incentivized to both collect representative information on drinking water safety and maintain staff with dedicated expertise in water quality testing.

Similar conclusions regarding the importance of motivational factors in other fields of development, including water and sanitation service provision, have promoted results-based financing mechanisms, which include output-based aid, provider payment incentives, performance-based financial transfers, and conditional cash transfers (Mumssen et al., 2010). In all of these models, achievement of pre-determined performance goals are recognized with financial or in-kind rewards. We suggest that comparable incentive strategies are important for motivating institutional leaders to establish the operating conditions required to meet monitoring performance targets.

The MfSW program did include pay-for-performance incentives, however, the effects of our incentives were not consistent across all MfSW collaborating institutions: i.e., some attained higher levels of performance than others for the reasons that we have documented. These variable outcomes emphasize that incentives alone are not always sufficient to overcome performance barriers. Optimally, efforts to strengthen local data collection and analysis, whether initiated by donor agencies, governments, or the monitoring institutions themselves should begin with a comprehensive evaluation all institutional conditions that determine performance. WaterCaRD, the scoring system that we developed for this study, provides a useful diagnostic for guiding this analysis. Subsequent interventions should then be customized to address relevant institutional gaps.

More broadly, we suggest that a similar gap analysis should guide “systems building” approaches that are increasingly discussed in the water and sanitation sector (USAID, 2014; Moriarty, 2016; Skilling, 2016; Rietveld et al., 2016). Proponents of systems approaches argue that discreet project-based interventions struggle to realize sustainable improvements in service provision because they generally do not identify and address all the factors that contribute to poor performance. Through the MfSW program we provide an example of how a system, in our case water quality testing, can be diagnosed to design evidence-based interventions that address critical weaknesses.
