# Supplementary material for: Why do water quality monitoring programs succeed or fail? A qualitative comparative analysis of regulated testing systems in sub-Saharan Africa
Source: Int J Hyg Environ Health. 2018 Jul;221(6):907–20. doi: 10.1016/j.ijheh.2018.05.010 (PMC6041725; doi:10.1016/j.ijheh.2018.05.010)
Supplement: Supplementary file 1 [file mmc1.docx]

**Supplementary Info**

**Table S1**: Qualitative data coding process and development of WaterCaRD Categories.

| **STEP 1** | **STEP 2** | **STEP 3** | **STEP 4** |
| --- | --- | --- | --- |
| DEDUCTIVE CODES  (Determined before 1^st^ round of coding) * | INDUCTIVE CODES  (Emerged during 1^st^ round of coding) | DEDUCTIVE CODES  (Determined before 2^nd^ round of coding**) **** | WATERCARD CATEGORIES*** |
| Institutional knowledge | Knowledge |  | **Staffing** |
| Personnel | Staff roles |  |  |
|  | Motivation |  |  |
|  | Staff stability |  |  |
|  |  | Staff recruitment |  |
|  |  | Risk management |  |
| Training | Training**^1^** |  |  |
| Oversight | Leadership |  |  |
|  | Enforcement |  | **Accountability** |
|  |  | National standards |  |
|  |  | Regulatory reporting |  |
|  |  | Consumer reporting |  |
| Program structure | Use of testing results |  | **Program**  **structure** |
| Testing set-up | Methods |  |  |
|  | Sampling plan |  |  |
| Transport | Transport**^1^** |  |  |
| Data use | Data management |  |  |
|  |  | Sample collection |  |
|  |  | Quality control |  |
|  |  | Remedial actions |  |
| Financial budget | Financial resources |  | **Finances** |
|  | Budgeting |  |  |
|  |  | Accounting |  |
| Equipment | Equipment |  | **Equipment**  **and services** |
|  | Maintenance |  |  |
|  | Procurement |  |  |
|  |  | Infrastructure |  |

* Most deductive codes were split into sub-codes during inductive coding (except training and transport); three deductive codes were not retained

** These were new deductive codes added for the second round of data analysis.

*** The 27 codes identified through the two rounds of analysis were sorted into the five WaterCaRD categories (see Table 3)

**^1^**These codes remained the same during inductive coding as no sub-codes were created

**Table S2:** fsQCA conditions and threshold calibration.

| **Condition** | **Scoring** |
| --- | --- |
| **Enforcement** | 0: Regulatory authorities (including direct line ministries) do not provide any feedback on reported data or do not exist.  0.33: Regulatory authorities provide some level of feedback on reported data, though there are no consequences (penalties or incentives) for the results.  0.67: Regulatory authorities provide some level of consequences (penalties or incentives) for results, though procedures may be informal or executed on an ad-hoc basis.  1: Regulatory authorities have procedures in place for formally incentivizing water quality data reporting or for penalizing poor reporting, and these procedures are regularly executed. |
| **Knowledge** | 0: Institution does not have staff with water quality monitoring experience.  0.33: Staff has some practical experience or theoretical knowledge of water quality monitoring, though this is limited.  0.67: Staff has comprehensive practical experience or theoretical knowledge of water quality monitoring, but not both.  1: Staff has comprehensive practical experience and theoretical knowledge of water quality monitoring (i.e., dilutions, multiple testing methods, data management, etc.). |
| **Motivation & Leadership** | 0: Institution is unclear on who is responsible for water quality management within the institution.  0.33: The institution acknowledges the importance of water quality monitoring, but leadership does not prioritize monitoring activities.  0.67: Institution's leadership prioritizes water quality monitoring but only some staff internalize this responsibility.  1: Institution's has a champion leader that drives water quality monitoring, and most staff internalize this responsibility. |
| **Staff Retention** | 0: Staff retention poses major challenges: there is lots of turnover and/or no processes in place to manage these transitions  0.33: Staff retention poses some challenges: there is some turnover and/or some gaps in processes in place to manage these transitions  0.67: Staff retention poses minor challenges: there is little turnover and/or most processes are in place to manage these transitions  1: Staff retention does not pose a problem to the institution: there is very minimal turnover and/or process are established to manage these transitions |
| **Transport** | 0: Institution does not have the means/transportation to collect samples and this is one of their main challenges.  0.37: Institution has transportation to collect sample but faces some serious transportation challenges.  0.67: Institution has transportation to collect samples, though faces occasional challenges such as vehicle availability or difficulty during the rainy season.  1: Institution has dedicated transport (such as a car or motorbike) available for sample collection. |
| **Equipment** | 0: Institution not very familiar with equipment distributors (prior to MfSW).  0.33: Institution has tried to contact equipment distributors but have faced substantial challenges obtaining what they need.  0.67: Institution has access to distributors, but there are some limitations to what they can get (e.g. limited product range, poor roads, long distances, etc.)  1: Institution has access to distributors and can obtain what they need. |
| **Procurement** | 0: Water quality manager or responsible persons are not familiar with institutional procurement procedures, or procurement procedures pose substantial challenges.  0.33: Water quality managers or responsible persons understand procurement procedures, but the procedures are not efficient and often pose challenges.  0.67: Water quality managers or responsible persons understand procurement procedures, and procedures sometimes pose challenges.  1: Water quality managers or responsible persons understand procurement procedures, and procedures are efficient and do not pose challenges. |
| **Infrastructure** | 0: Institution does not have dedicated space for water quality testing.  0.33: Institution has dedicated space for water quality testing, but the space is often lacking electricity.  0.67: Institution has dedicated space for water quality testing, but the space is sometimes lacking electricity.  1: Institution has dedicated space for water quality testing, which includes reliable electricity. |

**Table S3:** Data matrix of outcome and conditions for each case. Values in bold indicate high performance, defined as achieving at least the cross-over point of (i) 0.70 for Targets Achieved and (ii) 0.50 for Testing Consistency. Seventeen institutions met this criterion for Targets Achieved and ten institutions for Testing Consistency.

| **Code** | **Targets Achieved (Outcome**  **1)** | **Testing Consistency (Outcome 2)** | **Enforcement** | **Knowledge** | **Motivation & leadership** | **Staff retention** | **Transport** | **Equipment** | **Procurement** | **Infrastructure** |
| --- | --- | --- | --- | --- | --- | --- | --- | --- | --- | --- |
| E1 | 0.16 | 0.00 | 0.33 | 0.67 | 0.67 | 0.33 | 0 | 0.67 | 0.33 | 1 |
| E2 | 0.60 | 0.14 | 0.33 | 0.67 | 0.67 | 0.33 | 0 | 0.33 | 0 | 0.67 |
| E3 | **0.98** | 0.43 | 0.33 | 1 | 0.67 | 0.67 | 1 | 0.33 | 0.33 | 0.67 |
| E4 | **1.00** | **0.84** | 0.33 | 1 | 1 | 1 | 0.67 | 0.67 | 0.33 | 0.67 |
| G1 | **0.72** | 0.10 | 0.33 | 0.67 | 1 | 0.67 | 0.67 | 0.33 | 0.33 | 0.67 |
| K1 | **0.98** | **0.59** | 1 | 1 | 0.67 | 0.67 | 0.67 | 0.33 | 0.33 | 0.67 |
| K2 | **0.74** | 0.16 | 1 | 0.67 | 0.33 | 1 | 0.67 | 0.67 | 0.33 | 0.67 |
| K3 | **0.88** | 0.28 | 1 | 1 | 0.67 | 1 | 1 | 0.33 | 0.33 | 0.67 |
| K4 | **1.06** | **0.99** | 1 | 1 | 1 | 1 | 1 | 0.67 | 1 | 1 |
| K5 | 0.30 | 0.11 | 0 | 0.67 | 0.33 | 0 | 0.67 | 0.33 | 0 | 0.33 |
| K6 | 0.43 | 0.00 | 0 | 0.67 | 0.67 | 0 | 0.67 | 1 | 0.33 | 0 |
| K7 | **0.98** | **0.62** | 0 | 0.67 | 1 | 1 | 1 | 0.67 | 0.67 | 0 |
| S1 | **1.01** | 0.43 | 0 | 0.67 | 1 | 0.67 | 0.67 | 0.33 | 1 | 0 |
| U1 | **0.97** | 0.16 | 0.33 | 1 | 0.67 | 1 | 0.67 | 1 | 0 | 0.67 |
| U2 | **1.28** | **0.77** | 0 | 1 | 1 | 0.67 | 0.67 | 1 | 1 | 0.33 |
| U3 | **1.10** | **0.63** | 0.33 | 1 | 1 | 1 | 0.67 | 0.33 | 0.67 | 1 |
| U4 | 0.18 | 0.00 | 0 | 0.33 | 0.33 | 0.33 | 0.67 | 0 | 0.33 | 0 |
| U5 | 0.10 | 0.00 | 0 | 0.67 | 0.33 | 0 | 0.67 | 0 | 0 | 0 |
| U6 | **1.02** | **0.52** | 0 | 0.67 | 1 | 0.67 | 1 | 1 | 0.33 | 0.67 |
| U7 | 0.62 | 0.06 | 0 | 0.67 | 0.67 | 0.67 | 0.33 | 0.67 | 0.33 | 0 |
| Z1 | **1.43** | **0.68** | 1 | 0.67 | 1 | 0.67 | 0.67 | 0.33 | 0.67 | 0.33 |
| Z2 | **0.89** | **0.53** | 0.67 | 0.67 | 1 | 0.67 | 0.67 | 0.67 | 0.33 | 0.33 |
| Z3 | 0.64 | 0.34 | 0 | 0.67 | 0.67 | 0.67 | 0.67 | 0.33 | 0.33 | 0.67 |
| Z4 | 0.38 | 0.11 | 0 | 0.33 | 0.33 | 0.33 | 0.67 | 0.33 | 0.33 | 0.33 |
| Z5 | **1.22** | **0.74** | 0.33 | 0.67 | 0.67 | 0.67 | 1 | 1 | 0.33 | 1 |
| Z6 | **0.70** | 0.39 | 0.33 | 0.67 | 0.33 | 0.33 | 0.67 | 0.33 | 0.67 | 0.33 |
| *Median* | *0.89* | *0.37* | *0.33* | *0.67* | *0.67* | *0.67* | *0.67* | *0.33* | *0.33* | *0.67* |
| *Mean* | *0.78* | *0.37* | *0.33* | *0.75* | *0.72* | *0.62* | *0.68* | *0.53* | *0.41* | *0.49* |
| *Std Dev* | *0.36* | *0.30* | *0.38* | *0.20* | *0.26* | *0.32* | *0.26* | *0.30* | *0.29* | *0.34* |

**Table S4:** Consistency Scores for Targets Achieved (Outcome 1) and Testing Consistency (Outcome 2). Conditions with a consistency score of at least 0.9 are considered necessary.

| **Condition** | **Consistency Scores** | |
| --- | --- | --- |
|  | **Targets Achieved (Outcome 1)** | **Testing Consistency (Outcome 2)** |
| Knowledge | 0.90 | 0.98 |
| Motivation & leadership | 0.90 | 0.98 |
| Transport | 0.83 | 0.94 |
| Staff Retention | 0.82 | 0.94 |
| Equipment | 0.68 | 0.81 |
| Infrastructure | 0.63 | 0.73 |
| Procurement | 0.57 | 0.77 |
| Enforcement | 0.46 | 0.53 |
