# Supplementary material for: Why do water quality monitoring programs succeed or fail? A qualitative comparative analysis of regulated testing systems in sub-Saharan Africa
Source: Int J Hyg Environ Health. 2018 Jul;221(6):907–20. doi: 10.1016/j.ijheh.2018.05.010 (PMC6041725; doi:10.1016/j.ijheh.2018.05.010)
Supplement: Supplementary file 2 [file mmc2.pdf]

Needs Assessment – Interview Guide

Revised 9 March 2013

- Obtain:

Surveillance Agencies

a. Logbooks/Reports/Database of microbial tests,
b. Inventory of current testing equipment
c. Map of Area of jurisdiction (ideally with settlements)
d. Surveillance testing plan (if available)
e. # of sources in their area of jurisdiction
f. National standards

Suppliers

a. Logbooks/Reports/Database of microbial tests for 1 year
b. Inventory of current testing equipment
c. Sampling map (if available) (*whether as PDFs or JPEGs graphical formats or in GIS formats such as .shp or .mif files)*
d. Sampling plan –
e. # of connections and population for each scheme (Should already have this from applications – RP: we don't for suppliers managing multiple schemes)
f. WSPs
g. National standards

- Clarify: Any questions on application or logbook data received

To mention in introduction:

- We wish learn about more about ***your institution's constraints to microbial water quality testing, and why these constraints exist.***
- This is a **research project,** so we are gathering information on best to move forward. There are **many questions**, and some may seem repetitive, but the interview is structured to be very comprehensive. We are trying to **understand as much as we can about your institution**.
- The Needs Assessment is the next step before signing agreements with institutions. (***Describe agreement process and program timeline).***
- The interview ***will take at least 2 hours***, sometimes longer

- Everything you say is ***kept private***
- Please ***be as honest as you can***, and if there is anything you don't want to talk about, this is fine

******START RECORDING**

#	Main Questions – for all stakeholders	Managing Director probes	Technician probes
1	**Constraints/Capacity Building**<br>• Go through Written Needs Assessment, if available (otherwise, go through application requests)<br><br>**In your needs assessment/application you listed XYZ as major constraints or top priorities. Let's go into more detail. What specific outcomes would you like to see from the program?**<br>• Why are these inputs needed?<br>• Why do these gaps exist?<br>• Have there been attempts to address these gaps? (Integrate to other sections if possible). If they were not successful, why not?		
2	**Grand Tour**<br>If first visit: You have described your water testing program in the application. Please talk me through your testing program from the field to the lab.		
3	**Sampling Plan and Targets**<br>• How much and where do you sample?<br>o Are these fixed points or random?<br>• Do you have a specific sampling plan? If so, can you share with us?<br>• Location information:<br>o How do you record sampling	• Can we talk specifically about test numbers. In 2012, you report testing XXXX.<br>• Would you like to modify your sampling plan? If so, how?<br>o Do you want to do more tests at the same sites or add sites?<br>• What is your expectation for 2013 without	

#	Main Questions – for all stakeholders	Managing Director probes	Technician probes
	location information? GIS?<br>o Do you have a map of your sampling locations? Can you share with us?<br>o If your sampling points are part of a digital GIS mapping system, how have the sampling locations been recorded (i.e. using a GPS device or GPS-enabled phone or PDA?<br>o Does your institution have a GIS or digital mapping unit or specialist?<br>• How did you decide where and how much to test?<br>• I see from your application that you DO/DO NOT think that your current testing adequately covers your network/geographic area. Can you tell me more about this?<br>o What keeps you from adequately covering your network?<br>o Do you have targets for geographical coverage?<br><br>**What is the role of regulations or laws in deciding how much to test or where?**<br>• I see from you application that you ARE/ARE NOT meeting targets.<br>o What are your targets (if not provided)?<br>o What obstacles stop you from having more tests?<br>• Where do these targets come from (who makes the decision?)<br>• Are there specific laws, regulations or standards on water quality testing and results? Who are these published by? Are	the MfSW program?<br>• Is there a number you want to target in 2013/2014 with the support of this program?<br>• What information sources inform you how to improve your testing plan?	

The Aquaya Institute • 101 Larkspur Landing Circle, Suite 327 • Larkspur, CA 94939-1751 USA
www.aquaya.org • info@aquaya.org • +1 415 306 7593 (tel) • +1 415 306 7594 (fax)

#	Main Questions – for all stakeholders	Managing Director probes	Technician probes
	they enforced? By whom?<br>• If you are meeting targets, why do you want to participate in MfSW?		
4	**Sample Collection**<br>• Who collects samples?<br>• Who does the analysis?<br>• How are samples transported?<br>• What goes wrong or what are the challenges in this process? Why?		• What are the challenges of getting to some potential test sites?<br>• What is the furthest distance from sample collection to analysis site (estimate)?<br>• What is the length of time between sample collection and analysis?
5	**Staff Capacity**<br>• What are the roles and responsibilities of staff involved in water quality testing (if not clear from application)?<br>• If staff are currently collecting samples or using test results, but not actually doing the test itself, what is their capacity/knowledge in the area of water quality?<br>○ Do they understand the meaning of the different parameters?	• Please talk me through the staff's careers, from recruitment to training to job duties and compensation (for sampling and analysis)<br>• What are the full roles and responsibilities of these staff? (If need more detail than in application)<br>• Who do staff involved in testing directly report to (especially important for surveillance)?<br>• Which of the staff duties are more important than water testing (for those who collect samples, and those that test in lab)?<br>• Are there any performance incentives available for staff?<br>• What kind of training have staff involved in water testing or water quality management received?<br>• Are there specific skills you feel your staff lack?<br>• What type of training opportunities are available for staff?<br>• How often do staff receive training, and where does it happen?<br>• Is it a challenge to have enough skilled	• Please talk me through your careers, from recruitment to training to job duties and compensation.<br>• **Duties:**<br>○ What are your job duties in addition to water quality testing / managing?<br>○ Which of your other duties are more important than water testing? What type of training have you received related to water testing?<br>• **Training**<br>○ What training opportunities are available for you?<br>○ How often do you receive training, and where does it happen?<br>○ When did you last receive training?<br>○ If you are attend a training, what happens to your responsibilities?

#	Main Questions – for all stakeholders	Managing Director probes	Technician probes
		water testers?<br>• Are there dedicated funds for training?<br>• Let's talk about staff turnover. Is there a lot of staff turnover? Does staff turnover cause you any problems? Is there an initial training for someone new? Would this initial training involve water quality testing?	
6	**Equipment and consumables**<br>• I understand from your application that you use XX equipment, is this correct? (ask any clarifications here if necessary)<br>• How did you choose the equipment/method you are using?<br>• Any problems with current method or equipment?<br>• Do you have a written procedures/manuals or standard operating procedures (SOPs) for water quality testing?<br>• Does anything go wrong in the testing process?	• Where did you get the equipment you have? Where did the budget come from for this purchase (ie: internal budget or donor support)<br>• How did you decide which method/equipment to buy?<br>• Are there any problems with the testing method or equipment?<br>• Where do you source consumables?<br>• Are there in-country suppliers for the consumables required?<br>• Where do you source new equipment?<br>• If working with an external lab:<br>o How/why was this lab selected?<br>o Who owns the lab?<br>o Pricing for microbial testing<br>• Are there local rules and certification for certain testing methods or equipment? If so, by who makes these rules?<br>• If applicable, how did you come up with requests for the MfSW program? Did you already know what equipment was appropriate?	• More details on current microbial testing – manufacturer, broth used<br>o What are the strengths and weaknesses of this method?<br>• How is the equipment maintained?<br>• Do you have any equipment that is currently in need of repair? What has been done to repair it?
7	**Data Management**<br>• What happens to water test results? Who receives this information?<br>• What format are the results in?<br>• How long does delivery take?	• Is there someone dedicated to analyzing the data?<br>• What is done with the data? Communication to stakeholders, including users (public sharing)? Any examples?	• What follow up is done if water is contaminated? Any incident response protocol if contamination peaks?<br>i. Tell us about a recent

The Aquaya Institute • 101 Larkspur Landing Circle, Suite 327 • Larkspur, CA 94939-1751 USA
www.aquaya.org • info@aquaya.org • +1 415 306 7593 (tel) • +1 415 306 7594 (fax)

#	Main Questions – for all stakeholders	Managing Director probes	Technician probes
	• Is data regularly scanned or digitized? If so for what purposes?<br>• Are computer systems available onsite for scanning or to make use of spreadsheet programs? Is there an internet connection?<br>• Have other data management methods or software systems been considered? Is so, why are they not used?<br>• What would it take to let other stakeholders such as Customers, regulators, politicians, and so forth see these results?	• Are there people or organizations who might use water test results but do not receive them, or receive them late?<br>i. If so, what are the barriers to information flowing to everyone that might use it?<br>• Is there a way to recognize if the lab gave an inaccurate result?	experience when a water test showed contamination. What happened?<br>ii. What are the laws and regulations?<br>iii. What obstacles to you face for remediation when a problem is identified?<br>iv. Other stakeholders? Such as what happens if a regulator identifies contamination?
8	**Quality Control**<br>• Do you have a quality assurance plan for your testing activities?<br>• As far as you are aware, is there a lab accreditation process in this country?<br>• Is that something that you have considered or undergone?<br>• We will be going through a more detailed questionnaire checklist on quality control involving lab observations		
9	**Other Monitoring**<br>• Do you do sanitary inspections of your water system as part of your monitoring program (for both service provider and surveillance)? Can you describe what is included in these inspections. Is an inspection done every time a water sample is collected or are they done at independent intervals?<br>• Do you carry out any other monitoring type activities that you think we should know about?		

#	Main Questions – for all stakeholders	Managing Director probes	Technician probes
	• Surveillance: Do you monitor other parameters related to water service delivery?<br>o Prompt: Quantity, Accessibility, Affordability, Continuity		
10	**Use of Test Results**<br>• Tell me about the most recent time a water test showed contamination. Was there a response? If so, what was it? If no response, why not? Is this response or lack thereof typical?<br>• Are there any consequences if tests repeatedly show contamination? If so, what are the consequences?	• What happens if somebody omits a water test?<br>• How many days a month does the typical lab staff who is supposed to analyze water samples miss?<br>• How about those who collect water samples from the community?	
11	**Motivation**<br>• **Supplier: What is your overall perception of water quality in your system ?**<br>o Do people drink the water directly?<br>• Surveillance agency: What is your overall perception of water quality in your area of jurisdiction?<br>• Is having water quality data useful? Why?<br>• Is not having water quality data a problem?<br>• how does water testing fit into overall priorities?		
12	**Stakeholders:**<br>** NOTE: Ask any outstanding questions on the country framework here. Before interview look back at country frameworks to note where we still have questions.<br><br>**Can you describe how your institution fits into**	• Is the institution government or private?<br>• From your perspective, with regard to water quality testing, who are the other relevant stakeholders outside of your organization? What is your relationship with them?<br>• Do multiple institutions test the same	

#	Main Questions – for all stakeholders	Managing Director probes	Technician probes
	**the overall water service provision and water quality testing framework of the country? (If not fully understood)**	supply systems or point sources? If so, who are they?<br>• Whom do you report results to? Who reports to you, if any?<br>• What is your relationship with the water system users? How do you communicate, and how often? Are they informed of testing results?<br>• How do you log, address and monitor customer complaints?<br>• If applicable, how is the district health department structured?	
13	**Historical context**<br>• Describe history of the testing program –<br>o When did it take its current form? What was the situation previously?<br>o For suppliers, how old is the water treatment plant?		
14	**Financing:**<br>**We would like to understand more about your financing.**	• What are your revenue sources as a share of total revenue? How does total revenue compare with costs?<br>• Are there other sources of funding, since as government subsidies or donors?<br>• **If external tester: How much do you currently pay to process microbial samples?** How much would it cost to process 100 more samples a year?<br>o<br>• **If internal: How much would it cost to test another 100 samples for microbial contamination?** Can you break that cost down into supplies, transport, etc.?<br>• Regarding your budget, is there a specific line item for microbial testing? For total testing?	

#	Main Questions – for all stakeholders	Managing Director probes	Technician probes
15	**If time: Area context - Water quality and health**<br>• Water suppliers:<br>o What is the water source?<br>o What treatment processes are involved?<br>• Surveillance:<br>o Do you have a complete list of water sources in your district?<br>o If so, can we have a copy?<br>o Does this include the number of people using each point source?<br>• Health<br>o What is sanitation coverage like in this area?<br>o Are there any data records we could obtain on this?<br>o Is there any information on diarrheal disease, such as in children <5 years or for the whole population?<br>o Are there outbreaks of cholera in this area? Could we obtain information on cholera, giardia, dysentery infections?<br>• Are there census data and maps available of this area? If so, how would we obtain them?<br>• Can you describe the administrative structure of your area (i.e. Province, District, Subdistrict, Location, Sublocation, Village, etc.)? Is this information available?		

The Aquaya Institute • 101 Larkspur Landing Circle, Suite 327 • Larkspur, CA 94939-1751 USA
www.aquaya.org • info@aquaya.org • +1 415 306 7593 (tel) • +1 415 306 7594 (fax)
