# Supplementary material for: Why do water quality monitoring programs succeed or fail? A qualitative comparative analysis of regulated testing systems in sub-Saharan Africa
Source: Int J Hyg Environ Health. 2018 Jul;221(6):907–20. doi: 10.1016/j.ijheh.2018.05.010 (PMC6041725; doi:10.1016/j.ijheh.2018.05.010)
Supplement: Supplementary file 3 [file mmc3.pdf]

Date: ___________ Aquaya staff: _______________ Institution: ________________ Scheme/Region: ____________________Method observed: ___________________

OBSERVATIONS		Y/N (ea. sample)		
1	Sample Collection	1	2	3
	What type of water was being sampled? (tap, bh=borehole, ow=open well, sp=spring, sc=storage container, wtp, res=reservoir, rw=rain)			
1.1	For tap water: Was the tap sterilized (for suppliers) prior to sampling?			
1.2	Did the water flow before collection (for about 2 min)?			
1.3	Was a sterile sample container used for sample collection? **Take photo.**			
1.4	If water was chlorinated, was sodium thiosulfate used?			
1.5	Was the lip of the sample container touched with unsterile material or hands at any point?			
1.6	Were the samples labeled adequately (time, date, location/ID)? **Take photo.**			
2	Sample Transport			
2.1	Within how many hours are samples typically processed?			
3	Sample Storage and processing			
	Take Photos of Sample Processing and Equipment			
3.1	Were surfaces and hands sterilized/gloves worn prior to sample analysis?			
3.2	Was the volume used for processing adequate and measured precisely?			
3.3	Was the sample properly labeled? **Take photo.**			
3.4	Was the analysis conducted in a sterile manner (careful to not touching anything unsterile)?			
	Were equipment properly sterilized between samples?			
3.5	Was the incubator set to the correct temperature (37 for EC, 44 for TTC) or for Petrifilms, are they kept in a warm enough environment?			
3.6	Was media stored in the dark or made fresh?			
3.7	Are samples stored in a fridge or on ice if they are kept overnight?			
4	Lab environment			
4.1	Is lab space clean and separated from other work areas? List any concerns:			
4.2	Does staff readily have washing space and cleaning agent available?			
4.3	Does the lab seem regularly used (washed glassware drying, etc.)?			
4.4	Is sterilization of sample containers, petri dishes / test tubes, etc. adequate? List method:			
5	Data logging and management			
5.1	Were logbooks readily available when asked? **Take photo.**			

The Aquaya Institute

P.O. Box 5502
Santa Cruz, CA 95063
USA

Riara Corporate Suites, Suite #203
Riara Road, Kilimani Estate
Nairobi, Kenya

www.aquaya.org
info@aquaya.org
T +254.701.178.714

5.3	Did data appear to have been entered on different days and/or by different people?	
**QUESTIONS (ask in lab)**		
6	Overall	
6.1	What happens to the incubator when the electricity goes out?	
6.2	What is the calibration procedure for the incubator? Last calibrated (circle):<br>< 6 months 6mo-1 year >1 year Never	
6.3	If using MPN, do you have an MPN values table available?	
7	Lab evaluation / Accreditation	
7.1	Is there an external evaluation or accreditation process? If so what (such as ISO certification or checks by the Ministry)?	
7.2	If so, how often?	
8	Internal Quality Control	
8.1	Are there any internal checks by other staff members to ensure procedures are being followed correctly? Describe:	
8.2	(see table below)	
8.3	Have you ever had suspicious results or surprises?	
8.4	If so, describe why and what was done:	
**9**	**REFLECTION / PERCEPTION**	**Y/N**
9.1	Does sample collection and analysis seem adequate? If not, list why not:	
9.2	Does staff seem competent? If not, list why not:	
9.3	Does record keeping seem adequate? If not, list why not:	
9.4	How confident are we in their testing results? Provide an overall ranking of quality control (1-5). Explain:	
9.5	Did we run a duplicate sample and a positive or negative control?	
9.6	Were any concerns discussed? If so, describe:	

8.2:

Do you ever do	Y/N	Frequency (every batch, weekly, monthly)
• checks on media for sterility?		
• blank / negative controls?		
• duplicate or replicate samples?		

Other comments or concerns:
