# Supplementary material for: Why do water quality monitoring programs succeed or fail? A qualitative comparative analysis of regulated testing systems in sub-Saharan Africa
Source: Int J Hyg Environ Health. 2018 Jul;221(6):907–20. doi: 10.1016/j.ijheh.2018.05.010 (PMC6041725; doi:10.1016/j.ijheh.2018.05.010)
Supplement: Supplementary file 4 [file mmc4.pdf]

X Case Study

Table 1: Institution information and key dates.

Institution Information	Key dates and timeline for MfSW
**Agency**: Water Utility	27 Feb 13 - Application Received
**Regulator:** National Regulator	2 – 3 May 13 – In-Person Needs Assessment
**Population served**: 25,564	12 June 13 - Agreement signed
**# of Connections**: 913	1 Aug 13 - Upfront Funding Received
**Number of Water Schemes:** 2	29 Dec 13 - Finished Training
	1 Dec 13 - Testing Start Date
	6 Feb 14 - Meeting with X
	21 -22 July 14 - In-Person Midterm Assessment

Introduction

X is a water utility located approximately 345 kilometers by road from its country's capital city. The water utility serves 25,564 customers with 913 connections within its Municipality. X manages two water schemes, which include the main water distribution system and boreholes in one of the district's location.

Like many urban areas in the country, X is quickly growing, and, because of this, proper sanitation has become a problem in some areas. According to X's previous Managing Director (MD), open defecation is very common because of the high population growth rate. The institution's main water source, an open dam (constructed in 1982), is easily exposed to human and animal contamination as it is not properly fenced and secured.

Furthermore, there is also increasing building development in the vicinity of the water source. At the time of the needs assessment, the previous MD noted that a slaughterhouse was being built in close proximity to the dam and plans were underway to build a commercial center nearby. To prevent this, the institution collected data on the negative effects of building a slaughterhouse near a municipal water source, which advised local authorities on whether they should allow the construction to continue. In the end, the local authorities discontinued the slaughterhouse construction.

In the X's country, the national government is increasing decision-making authority to local governments, which has had mixed results to date across the country. At X, however, it has largely been seen as a positive move. This decision by the local authorities to ban the construction of the slaughterhouse marked one of the benefits that X derived from this process. Other positive impacts include:

- Timely payment of operational costs of the treatment plant by the local authorities. Water pumping hours are no longer reduced because of delayed operational cost payments, which was the case when the institution was under the national jurisdiction.
- A vehicle and motorbikes were donated to X by the local authority funds.
- Previously, the institution paid a 9% levy from their total revenue to a regional water services board.
- According to current MD, X did not see any benefits from this levy. However, the local authorities have provided a lot of support to the institution without imposing levies.

During the midterm assessment, the MD also noted that the institution had a stakeholder meeting during MfSW where they discussed the possibility of expanding the institution a broader geographical area beyond their municipality.

1. Accountability

1.1. National standards

As a water utility in the country, X reports to the national independent water regulator, a non-commercial state corporation mandated to oversee the implementation of policies and strategies relating to provision of water and sewerage services (more in section 2.2). The regulator has set specific guidelines for water

quality monitoring, in line with the national Bureau of Standards. X operates as an agent of the regional water services board to provide water and sewerage services to the residents of the town, which falls under the town council. Due to increased responsibility to local governments, however, the regional water services board is likely to be phased out and X will start reporting to local government authorities.

1.2. Regulatory Authority

The MD of X sends quarterly summary reports to the national independent water regulator on the percentage of water tests in compliance (the proportion of negative microbial test over microbial water tests conducted).

1.3. Consumers

X does not share microbial water test results with their users. They do, however, communicate with the public when conducting microbial water sampling by word of mouth. During sampling, customers communicate with the staff about the water they receive from the institution and any challenges they face regarding water. According to X's Technical Manager, they made a public announcement by radio and word of mouth at the start of the MfSW program that they would be performing routine water sampling and testing. Prior to MfSW, the Technical Manager noted that they communicated X's water quality and rationing schedule every two months via the local radio. During the midterm assessment, the Technical Manager noted that since they started conducting testing, the consumers perceived the testing as a good activity, which has acted as extra motivation for X's staff.

1.4. Enforcement

The institution receives annual feedback on their performance (established from the summary reports) from the regulator through their annually published reports. The report documents the performance of all water utilities in the country across a wide range of indicators and serves to benchmark utility performance against one another.

2. Staffing

2.1. Leadership

The institution's microbial water testing program seems to be well managed. The current MD provides general oversight and the Technical Manager is the direct manager of the testing team.

The current MD is the MfSW point person. During the initial phases of MfSW, the previous MD left the institution and was replaced by the current one who, at the time of conducting the midterm assessment, was the acting MD. Before becoming X's formal MD, the current MD was the institutions' Accounting Officer. During the midterm assessment, an Aquaya staff noted that it seemed "like [the previous MD] performed an adequate handover to [the current one], because [the previous MD] was aware of the MfSW program and supported it" (midterm assessment, 2014).

During the midterm assessment, the current MD noted that he was exploring how to continue microbial water testing beyond MfSW by including monitoring in the institutions' normal budget as part of their operations. The current MD and Technical Manager demonstrated to provide support to the lab/plant team as they normally would.

2.2. Staff Roles

The institution had six employees that were primarily involved in MfSW. X had two laboratory technicians responsible for microbial water sample collection and testing, who were also the plant operators. During the MfSW program, the institution worked with an intern, who according to the current MD, was soon to be hired because he was young and motivated.

The staff roles were as follows:

- A: the initial MD and point person; sent monthly microbial water test result data to Aquaya and compliance data to the regulator.
- B: The current MD who replaced the initial MD mid-program.
- C (X's Technical Manager): Oversaw lab staff and operations; determined the follow up actions to be taken when contamination was detected in X's water supply.
- D and E (X's plant operators and laboratory technicians): Conducted microbial water sampling and testing.
- F (intern at X at the time of the midterm assessment): Assisted the plant operators and lab technicians with microbial water sampling, testing, result interpretation, and recording results. During the midterm assessment, the current MD noted that the institutions' lab technician (D) was going to retire soon and that F, the intern, would take his position.

2.3. Knowledge

Prior to MfSW, X used the most probable number (MPN) multiple test tube method to conduct microbial water testing. The staff had not received any formal training on microbial water quality testing or monitoring and solely relied on an outdated manual. During the needs assessment, the lab staff noted that they had microbial water testing equipment that they did not know how to use. During MfSW, staff were trained on using membrane filtration (MF) for microbial water testing because X wanted to "switch to this method to get a more accurate understanding of the contamination levels" (midterm assessment, 2014). One of the plant operator's and laboratory technician has over 30 years' experience as a lab technologist and plant operator and was trained on water quality and pollution control at the time of conducting the needs assessment. The other plant operator and laboratory technician completed his A-level. Before becoming the lab technician and plant operator, he was a Chemical Attendant at X. The Technical Manager at X has a Degree in Water and Environmental Engineering and has worked for the institution since 2009.
Staff have integrated and adapted the MfSW program into their normal routine. Since the technical team had previous experience with quantitative microbial testing, they were able to leverage this experience when designing their testing activities under MfSW.

2.4. Training

Four employees from X received a four-day training on microbial water testing and monitoring using MF method. A Technical Manager from Z, one of the MfSW partner institutions, facilitated the training. The previous MD chose to have the Technical Manager from Z conduct the training because he knew him when he was an intern at Z. X initially wanted to attend training at an Institute in the capital city but found the training too expensive and therefore asked the Technical Manager from Z to facilitate the training. The MD believed the Technical Manager's knowledge of the MF method was sound, the two had a good working relationship, and the fee was consistent with the MfSW budget. X chose to be trained on MF because they wanted to have a better quantitative understanding of the contamination levels of their water as compared to the MPN method. After the training, the staff noted that they were more confident in their abilities to conduct microbial water monitoring and testing.

2.5. Motivation

X is a small enterprise and staff demonstrated a sense of pride and ownership in their growing business. This likely helped the overall motivation levels at the utility. X is the only MfSW partner water utility institution in the country that provided its lab staff with extra incentive, on top of the staff monthly salaries, from the MfSW per test payment to conduct microbial water testing. The institution received 20 USD per test for microbial water testing from the MfSW program and also provided their staff with per diem for sample water collection and testing. However, Aquaya does not know what impact this had on the microbial water testing program on the institution.

During the midterm assessment, the current MD noted that the MfSW program had been beneficial to the institution. He noted that a Bureau of Standards from the country had evaluated the institution and given

them a positive feedback. The Technical Manager added that the institution was also able to host an intern because they had the capacity to do so from the MfSW program.

During the midterm assessment, an Aquaya staff observed that, generally, the laboratory staff seemed committed to the microbial water testing program. It is also important to note that the current MD has been timely in the submission of microbial water test results to MfSW through the Aquaya Institute. The Aquaya staff also observed that the technical manager was enthusiastic about the MfSW program.

2.6. Staff Stability

During the MfSW program, X had one staff transition. The previous MD left the institution and was replaced by the current one. The previous MD was transferred to the local government where he is now the Chief Officer of Transport and Public Works. This staff transition did not seem to affect the program. In fact, it was perceived that the transition might be beneficial in terms of receiving continued support from the local government authorities.

2.7. Staff Recruitment

Staff transitions at the institution mostly seemed to be a result of promotions. For example, before becoming X's formal MD, the current MD was the institutions' Accounting Officer. The previous MD was then transferred to the local government where he is the Chief Officer of Transport and Public Works. During the midterm assessment, X's current MD noted that the institution was planning on employing a current intern to replace one of the Plant Operator and Laboratory Technicians who was planning on retiring. The institution provided the intern with microbial water testing and monitoring training during the MfSW program. Before becoming a Plant Operator and Laboratory Technician, one of the staff was a Chemical Attendant at X.

2.8. Risk Management

The institution faced the following challenges as part of their microbial testing program:

- The previous MD was transferred to the local government: The institution made the Accounting Officer the acting MD. Currently, the acting MD is now the institution's MD. This did not seem to interfere with the water quality monitoring and testing program.
- The institution's water monitoring equipment occasionally broke: Monitoring and testing was suspended for several days until the equipment was replaced or repaired.
- Frequent electricity power cuts interfered with the testing program since the institution relies on electricity to supply water: The institution suspended testing when this happened since they did not have electricity back-ups for power outages.
- The institution's main water supply (a dam) dried up in March 2015: X did not conduct water testing and could not supply water to consumers because they primarily relied on the dam for water. During the midterm assessment, the current MD noted that the local government was planning on constructing a new dam. According to him, the dam was going to be located on higher ground and would use gravity to supply water. This would be economically advantageous for the institution and it would also mean that X's customers would be able to get water even when there were power cuts. However, Aquaya does not know if this happened.

3. Program Structure

3.1. Methods

X conducted microbial water testing using MF during the MfSW program. During the midterm assessment, the following (Table 2) was observed during sample collection and testing:

Table 2: Testing Activities.

Activity	Comments
A sterile sample container was used for sample water collection.	This is a good testing practice and water test results would not be compromised.
Hands were sterilized with methylated spirit instead of using gloves	This is a good practice.
Sterilization of sample containers, petri dishes, and test-tubes was done adequately.	This is a good practice. Sample containers, petri dishes and test-tubes should be sterilized before testing.
Media was improperly sucked up using a pipette by the mouth of a technician because they lacked a suction pump.	This is not a good practice because microorganisms from the mouth could enter the media and interfere with the test results.
Sodium thiosulphate was improperly used on the filter paper; 10 ml of sterile water is run through the filter paper and then sodium thiosulphate ran through the same filter paper.	This is not the right testing procedure. Sodium thiosulphate is supposed to be added to the water sample before testing to inactivate any traces of chlorine in the water. This practice could potentially interfere with the microbial water test results.

3.2. Use of Testing Results

X conducted negative controls for every sample collected during the MfSW program. X recorded high contamination levels during the rainy seasons. According to the current MD, "during the rainy season raw water is highly contaminated, [and] testing with MfSW has made us realize just how much contaminated [the water] can get" (midterm assessment, 2014). The institution also found contamination in their consumer water storage tanks. During the midterm assessment, the Technical Manager noted that whenever they take samples from their consumer water storage tanks, they "see small things moving in the sample" (midterm assessment, 2014). According to the Technical Manager, many of their consumer "tanks can stay for more than three years without being used or cleaned" (midterm assessment, 2014). However, according to him, positive water test results from the institution's distribution system are very rare and the institution has only received one consumer complaint about water quality since he started working for the institution.

3.3. Sampling plan

X decided on an annual test target of 312 for the MfSW program. The institution collected and tested six samples per week (two samples from raw water, two from the treatment works, and two from the distribution network). Their rationale for this sampling plan was that they wanted to see the bacteria levels in different parts of the water supply system and because "the catchment of the dam is not protected from human settlement and 77% of the [town's] population living in the catchment practices open defecation" (application, 2013). According to the World Health Organization (WHO) Water Quality Testing Guidelines, the institution is responsible for conducting 56 microbial water tests per year based on their population size. The national regulator requires 12 tests per year for a utility of X's size. X's sampling plan therefore exceeded both the WHO and the national regulator's test targets. During the midterm assessment, an Aquaya staff noted that the institution also had a written sampling plan that was displayed in the lab.

3.4. Sample Collection

During the midterm assessment, the Technical Manager noted that their MfSW testing targets were adequate. He also mentioned that the major challenges that prevented the institution from reaching their test targets were:

- Xs' equipment breaking on occasion. For example, their water pump broke and the institution could not supply water and was therefore unable to collect water samples. In January 2015, water was highly turbid because of the rainy season and their turbidimeter broke. In an email to Aquaya, the current MD explained that the reason the institution could not reach test targets was because

"during the last days of sampling, the turbidimeter became inaccurate due to [excess] dirt" in the raw water (reasons for testing or not testing, 2015). However, it is important to note that X's MF test kit for microbial water testing was in good condition. The turbidimeter only tests for water turbidity.

- In February 2014, X did not reach their test target because "February ended early (28 days) so they did not meet their targets based on their sampling plan since they only test six times per week" (midterm assessment, 2014).
- Electricity (power cuts): "If there is no electricity, there is no water, so we cannot test" (midterm assessment, 2014).

During the midterm assessment, X's hand pump used during membrane filtration broke (Figure 1) and testing of the collected water samples had to be postponed to the following day.

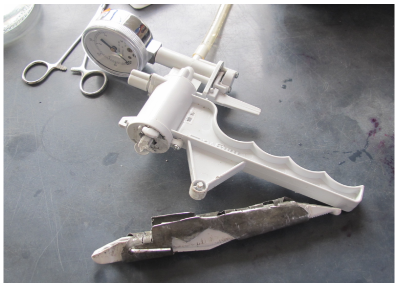

Figure 1: A broken hand pump at X.

3.5. Transport

X's catchment area is relatively small, but the terrain is very hilly and the roads are in poor condition. Before MfSW, the staff had to walk to the sample collection sites to collect water samples. During the needs assessment, the previous MD noted that "plant operators and technicians currently walk to sampling points to collect samples, which is very time consuming" (Needs assessment, 2013). The institution occasionally hired motorbikes or vehicles for sample transportation when needed.

Using the MfSW transportation start-up funds of 1,000 USD, X was able to hire motorbikes for sample transportation. When asked during the midterm assessment what had enabled them to reach their test targets, the current MD said the main reason was because their transportation needs were met. The staff used the transportation start-up funds from MfSW to hire motorbikes to use for sample transportation.

3.6. Quality Control

X conducted negative controls for every sample collected during the MfSW program, based on Aquaya's recommendations. It should be noted that they were the only MfSW partner institution to do so.

3.7. Data management

Before MfSW, X's laboratories did not have a computer to record water quality testing data. After testing, results were manually recorded on a piece of paper by the laboratory technician. Later, the technician scanned the papers and sent them to the MD. The MD then sent quarterly reports to the national regulator, which included the number of microbial water tests conducted. The institution also had an operators book, which contained summaries of the water tests conducted by the institution.

During MfSW, the institution moved one of the computers that was in X's main office to the laboratory. An Aquaya staff noted, "they have now provided the lab with a computer from the office and a modem (so they can send emails to the MD), although they are currently just copying results onto a USB drive and bringing it to the main office" (midterm assessment, 2014). During the midterm assessment, the current MD noted that one of the reasons the institution was able to consistently send testing data to Aquaya during the MfSW program was because of improved communication between the lab and the main office.

During the program, information was communicated as follows:

- The lab technologist recorded the microbial water test information (sampling location, sample type, test conducted) and results on a piece of paper.
- The lab technologist transferred this information to Excel on the laboratory computer.
- The staff then saved the information on a USB drive and brought it to the Technical Manager in the main office.
- The Technical Manager examined the Excel files and the raw data at least twice per week, and if there was contamination, he determined the necessary follow-up actions.
- The Technical Manager then brought the test results to the MD.
- The MD sent monthly water test results (in Excel format) to Aquaya as well as a monthly report on the number and type of water tests conducted to the regulator.

3.8. Remedial Actions

When discussing the follow-up actions taken by X following contamination, it is important to note that the institution rations their water in different parts of the town at different days of the week. According to the MD, this is because "we don't have enough water to service the entire town, especially during dry season. We reduce service hours from 22 hours to 18 hours—this is the amount of time we are pumping the water through the water treatment plant to the storage tanks throughout the city. The dam cannot sustain the population of town X which has been growing" (midterm assessment, 2014). Some parts of the town, which rely on the institution's water supply, might be without water for up to four days in a week.

Because of this, most of X's consumers have invested in purchasing water tanks to store water they receive from X. The tanks are also connected to consumers' rooftops so that they can collect rainwater. Rainwater, therefore, sometimes mixes with X's treated water, and, as a result, some of the consumers have contaminated water. During the midterm assessment, the Technical Manager noted that most of the consumers used rainwater which is often contaminated due to the dust accumulated on rooftops. Whenever the institution collected water samples from such consumers, they advised them "to use water filters to combat household contamination" (midterm assessment, 2014). They also advised their consumers to clean their storage tanks on a regular basis to prevent water contamination.

Other follow-up actions included increasing chlorination levels and advising their users on how to ensure that their water was safe for drinking. Whenever contamination was detected in the distribution system after testing, X increased the level of chlorine in the water.

During the midterm assessment, the current MD noted that the institution does not communicate microbial water test results to their users. However, before the MfSW program, the institution communicated information about water rationing, and sometimes water quality, through the local radio station, which they no longer did during the MfSW program.

4. Finances

4.1. Financial Resources

Besides the MfSW funds, the institution had never received any financial support from any other organizations. X received the following from MfSW as start-up funds:

- Equipment: 2,861 USD
- Training: 882 USD
- Transportation: 1,000 USD

4.2. Budgeting

The institution demonstrated proper budgeting of their MfSW start-up funds. The institution provided their staff with per diem as incentive. Aside from this, the institution allocated the MfSW transport start-up funds to transportation needs, unlike some MfSW partner institutions. During the midterm assessment, the institution was able to account for all the funds that they used from the MfSW program. Prior to MfSW they did not have a specific budget for water quality monitoring. During the midterm assessment, the current MD noted

that the institution was planning on incorporating the Aquaya program into their normal budget as part of their operations when the program ended in December 2014.

4.3. Accounting practices

X has been timely in the submission of receipts to the Aquaya Institute; X submitted most of their receipts for the MfSW program. Aquaya does not have detailed information on their accounting practices.

5. Equipment and Services

5.1. Equipment

X's location far (345 kilometers by road) from the capital city, put the institution at a disadvantage in terms of accessing quality microbial water testing equipment. Much of the equipment purchased by the institution was done locally within the region. However, compared to the capital city, equipment is more expensive and/or of poorer quality in the region. X purchased filtration units, other consumables, and a vacuum pump for MF testing in the capital city, as these MF-specific items could not be found locally.

5.2. Maintenance

X does not have an equipment maintenance system. Before MfSW, they had several pieces of equipment that were not working. For example, their chemical stirring equipment (not for microbial water testing), according to the previous MD, was "not up to par at the treatment plant" (needs assessment, 2013). The previous MD mentioned that the stirring equipment would occasionally cause problems with water quality since chemicals had to be manually added, so measurements were sometimes off. The institution also had a water distiller that had not been connected at the time of the needs assessment. During the midterm assessment, the water distiller was still not connected. The institutions' hand pump used for MF also broke during the MfSW program, which, according to staff, was very challenging to replace as it was a special part that could only be ordered through certain suppliers in the capital city. As a result, testing was postponed to the following day when the pump started working again. Aquaya is notes that equipment and supply maintenance did not seem be a significant barrier to testing.

Before MfSW, X had several challenges with its equipment. They had a water distiller that was not functional and no one seemed to know how to install. They also had an incubator that could only hold a limited number of samples. X's MPN multiple test tube set-up was adequate, but glassware was insufficient and outdated.

5.3. Procurement

To procure microbial water testing equipment under MfSW, the institution used their normal procurement process, which involved the following steps:

- X had a pre-qualified suppliers list, which was revised annually. Equipment quotes were requested from these suppliers.
- The institution received four quotations from four suppliers.
- Out of the four suppliers, the institution selected two suppliers who had the lowest prices for equipment.
- Local purchase orders were developed to order equipment.
- X then sent payments to the suppliers after receiving an invoice.
- Equipment was delivered with a delivery note.

The vacuum pump, however, could not be purchased from any of their pre-qualified suppliers, so they had to single source this item. The Technical Manager, therefore, went to four shops in the capital city and finally purchased the vacuum pump from a lab supply center in the capital city.

5.4. Infrastructure

The road infrastructure in the town is sub-par. The majority of roads in and around the town are unpaved. In fact, the main road to reach the town from the capital city is paved only up to about halfway. It takes approximately 7-9 hours to reach the town from the capital city, and much of this time is spent on a road only accessible by 4x4 during the rainy season. This, however, did not seem to interfere with microbial water testing as the institution mainly used motorbikes for sample transportation. During the midterm assessment, the current MD noted, “since we now hire motorbikes, we can now access water sources better in the area” (midterm assessment, 2014).

X’s laboratory, located at the treatment plant, has sufficient workspace for testing, but the building and much of the equipment is old and needs to be repaired and updated, which is most likely due to the town’s isolation from the country’s main arteries.

A factor that hindered microbial water testing were the electrical power cuts that sometimes occurred in the town. When asked what factors hindered X from meeting their test targets, the Technical Manager noted, “if there is no electricity, there is no water, so we cannot test” (midterm assessment, 2014).

According to the current MD, the local government was also planning on potentially building a new dam to address the inadequacy of the X dam. A staff member noted that with the new dam, microbial water monitoring would likely to increase at the institution.

Table 3: Situational analysis (before and after) MFSW- what changed?

**Category**	**Before MfSW**	**After MfSW**
Transportation	Lab technicians walked to the sampling points to collect water samples.	X hired motorbikes to use for sample transportation
Staffing	The staff had not received any training on microbial water quality monitoring and testing; they only relied on dated manuals on the MPN method.	The staff received training on MF<br>• Staff seemed committed to microbial water quality monitoring

Table 4: MfSW Funding requests.

	**Initial Requests (written NA)**	**In agreement**	**Actual**
Equipment	Colony Counter plates<br>File bottles<br>Incubator<br>Autoclave<br>Cooler box	Membrane filtration equipment<br>Multiple test tube equipment	Equipment primarily for membrane filtration, most of which was procured in September 2013; vacuum pump took a bit longer and was procured in October 2013
Transport	Vehicle or motorbike	1,000 USD motorbike hire and fuel allowance	1,000 USD motorbike hire and fuel allowance
Training	Comprehensive training starting with sampling; no specifics were given	4-day training focused on multiple test tube and membrane filtration methods. 2 lab technicians participated	4-day training focused on multiple test tube and membrane filtration methods. 2 lab technicians participated

Data Management	Computers and software requested	Not funded	NA
Other	Workshop with community	Not funded	NA

The Aquaya Institute

P.O. Box 5502
Santa Cruz, CA 95063
USA

Riara Corporate Suites, Suite #203
Riara Road, Kilimani Estate
Nairobi, Kenya

www.aquaya.org
info@aquaya.org
T +254.701.178.714
