# Supplementary material for: Why do water quality monitoring programs succeed or fail? A qualitative comparative analysis of regulated testing systems in sub-Saharan Africa
Source: Int J Hyg Environ Health. 2018 Jul;221(6):907–20. doi: 10.1016/j.ijheh.2018.05.010 (PMC6041725; doi:10.1016/j.ijheh.2018.05.010)
Supplement: Supplementary file 5 [file mmc5.pdf]

Midterm Assessment – Interview Guide
22 May 2014

To obtain

- Receipts for anything missing from upfront grant or per-test payments
- Financial records – last year's and this year's (and year after the program)
- Written needs assessment (if did not submit before visit)
- Post-training evaluation – send ahead of time
- Training reports, if missing
- Photos of logbooks
- Sanitary survey scale, if available

To mention in introduction:

- We wish to:
  - ***check up with you on the MfSW program and see how everything is progressing***
  - ***understand in more detail why you are meeting/not meeting targets***
  - ***if there are remaining challenges to water quality testing, and***
  - ***discuss what, if anything, might have been done differently with MfSW in order to meet these targets.***
- ***We do not plan to provide additional resources at this stage***, though we will be asking about training & equipment and existing gaps. This is a research project, and the objective is to understand the program as best we can.
- The interview ***will take at least 2 hours***, sometimes longer. We ***would like to conduct some samples and tests*** with you so it might be possible to conduct some of the interview during this process
- Everything you say is ***kept private***
- Please ***be as honest as you can***, and if there is anything you don't want to talk about, this is fine

******START RECORDING**

#	Ask questions to the person that manages the testing program
1	**General**<br>– Talk me through your institution's MfSW progress to date.<br>o Effects of the program (good or bad)<br>o Changes<br>o Challenges<br>o Unique things you have encountered
	**MfSW Program Set-up**
2	**Equipment procurement**<br>– Talk me through the equipment procurement process, from determining what you needed through to today.<br>o Tell me about the suppliers you chose. Who were they, where were they, and how did you select them?

The Aquaya Institute
P.O. Box 5502
Santa Cruz, CA 95063
USA
Riara Corporate Suites, Suite #203
Riara Road, Kilimani Estate
Nairobi, Kenya
www.aquaya.org
info@aquaya.org
T +254.701.178.714

	– How did the procurement for MfSW compare to your normal procedures?<br>o What was the approval process like?<br>– How did the actual purchasing of items compare to what was in the funding plan (some items not purchased, or additional items purchased? If there were changes, why? How was that decided?
3	**Training**<br>– Can you talk us through the training process (from organizing to attending)?<br>o Who led training?<br>▪ How were they selected?<br>▪ Did they seem competent?<br>o Where did the training take place?<br>o Who attended the training?<br>▪ Anyone from external institutions?<br>o How was the approval process?<br>– What are the outcomes or results of the training? What has the training achieved?<br>o How confident are you in your/staff's ability to collect and analyze samples?<br>– How did the training compare to the original plan in the agreement (review)?<br>– Ask about post-training evaluation – anything that came up<br>– Ask for training reports, if missing
4	**Staff management**<br>– Can you talk us through the management of the program? Who is involved and what are their roles?<br>o How were these staff decisions made?<br>o What were their roles prior to MfSW?<br>– Have you had any challenges regarding staff management?<br>– Have you had any staff changes since the start of the program? If so, what? How has that affected the program?<br>– How does MfSW fit into your other priorities and other activities of the institution?
**MfSW Implementation Phase**	
5	**Testing Targets**<br>– How is your progress on meeting testing targets? (review)<br>o What factors enable you to meet targets?<br>o What factors hinder you from meeting targets?<br>– Do you think the testing targets (number of samples and locations) are adequate? Is there anything you would change?
6	**Perception of water quality**<br>– How is the water quality in your area? How much contamination are you seeing?

The Aquaya Institute
P.O. Box 5502
Santa Cruz, CA 95063
USA
Riara Corporate Suites, Suite #203
Riara Road, Kilimani Estate
Nairobi, Kenya
www.aquaya.org
info@aquaya.org
T +254.701.178.714

	o Have the results from MfSW been surprising? Why or why not?<br>– Since the MfSW program inception, have there been any changes in management of water quality within your institution?<br>– Do most people in the area drink this water directly?<br>o Do people treat their water at their home?<br>o What water do you drink at home? (review answer from needs assessment)
7	**Data sharing**<br>– What water quality data do you see (raw data, summary, report)? How often? How do you get it (email, going to the lab)?<br>o Do you look for contaminated samples? When? What do you do if you see one?<br>o Do you look at trends in the data (which samples are contaminated)?<br>– Where do you send the testing results besides Aquaya? What type of data do you send (raw data, number of tests conforming, etc.)? How do you compile this (raw logbook, excel?)<br>o If shared with the regulator or ministry, have they provided any feedback?<br>▪ Have there been any recent changes in regulation or enforcement? Explain<br>▪ Have there been any decisions about infrastructure (e.g. upgrading water treatment plant) because of these reports?<br>▪ (Review enforcement / institutional framework and clarify if necessary)<br>– Is information on testing results communicated to the users or the general public?<br>o If yes, how (physical posting of data, online information, ministerial reports, etc)?<br>▪ What is the response from users?<br>o If not, if this something you could consider doing? Why or why not?<br>▪ Is this something that users have asked about?
8	**Financials**<br>– How have you spent the per-test payment(s) you have received to date?<br>– Do you receive additional funds, outside of MfSW for water quality testing?<br>– In your application, you stated that your water quality testing budget was XX. How is this budget determined?<br>o How does this budget compare in 2012, 2013, 2014? Can you provide us with a breakdown? (Ask to explain the variation)<br>– Ask for receipts if anything is missing
	**Clarify Any Other Questions**
9	– Any data on sanitation/health?<br>– Anything from case study?
	**Closing Questions**
10	**Closing**

The Aquaya Institute
P.O. Box 5502
Santa Cruz, CA 95063
USA
Riara Corporate Suites, Suite #203
Riara Road, Kilimani Estate
Nairobi, Kenya
www.aquaya.org
info@aquaya.org
T +254.701.178.714

	– Looking back on the program, is there anything you would have done differently or would like to change?<br>– What do you predict will happen after the program ends?<br>o If you plan to continue the program, how will these costs be covered?<br>o If you plan to continue the program, would you make modifications to the program (testing targets, sampling plan, etc)? If so, what?<br>– Quality control: Explain quality control (positive control, negative control, replicate). Explain we'd like to ask them to start doing some samples.<br>o If they're not using sodium thiosulfate, ask if they can start using it.<br>– Is there anything else you would like to tell us? What else should we know?<br>– Is there anyone else we should speak to about the program?<br>– Do you have any questions for us?<br>– Next Steps<br>o Primary aim of MfSW<br>▪ As a reminder this is a research project with the primary aim of analyzing the constraints institutions like yours face to microbial water quality monitoring<br>▪ Long term extension or scaling up of the program is not an option<br>▪ The aim is for the findings from this study to push the agenda of water quality monitoring and ultimately promote effective policy change to sufficiently address monitoring needs<br>o That said, testing will go until October of this year (to amend based on institution) after which we will:<br>▪ Collect additional testing data for six months<br>▪ Conduct endline surveys, where necessary<br>▪ Write reports and publications<br>▪ Present findings to local and international groups<br>▪ Organize forums and workshops with partner institutions<br>▪ We will communicate the details of this final portion of the study as things come into focus

The Aquaya Institute
P.O. Box 5502
Santa Cruz, CA 95063
USA
Riara Corporate Suites, Suite #203
Riara Road, Kilimani Estate
Nairobi, Kenya
www.aquaya.org
info@aquaya.org
T +254.701.178.714
